# Evaluating Biparametric Versus Multiparametric Magnetic Resonance Imaging for Diagnosing Clinically Significant Prostate Cancer: An International, Paired, Noninferiority, Confirmatory Observer Study

**DOI:** 10.1016/j.eururo.2024.09.035

**Published:** 2024-10-22

**Authors:** Jasper J. Twilt, Anindo Saha, Joeran S. Bosma, Bram van Ginneken, Anders Bjartell, Anwar R. Padhani, David Bonekamp, Geert Villeirs, Georg Salomon, Gianluca Giannarini, Jayashree Kalpathy-Cramer, Jelle Barentsz, Klaus H. Maier-Hein, Mirabela Rusu, Olivier Rouvière, Roderick van den Bergh, Valeria Panebianco, Veeru Kasivisvanathan, Nancy A. Obuchowski, Derya Yakar, Mattijs Elschot, Jeroen Veltman, Jurgen J. Fütterer, Henkjan Huisman, Maarten de Rooij, PI-CAI Consortium

**Affiliations:** a Minimally Invasive Image-Guided Intervention Center, Department of Medical Imaging, Radboud University Medical Center, Nijmegen, The Netherlands; b Diagnostic Image Analysis Group, Department of Medical Imaging, Radboud University Medical Center, Nijmegen, The Netherlands; c Department of Urology, Skåne University Hospital, Lund, Sweden; d Division of Translational Cancer Research, Lund University Cancer Centre, Lund, Sweden; e Paul Strickland Scanner Centre, Mount Vernon Cancer Centre, Northwood, UK; f Division of Radiology, Deutsches Krebsforschungszentrum, Heidelberg, Germany; g Department of Diagnostic Sciences, Ghent University Hospital, Ghent, Belgium; h Martini Clinic, Prostate Cancer Center, University Medical Centre Hamburg-Eppendorf, Hamburg, Germany; i Urology Unit, Santa Maria della Misericordia University Hospital, Udine, Italy; j Division of Artificial Medical Intelligence in Ophthalmology, University of Colorado, Boulder, CO, USA; k Department of Medical Imaging, Andros Clinics, Amsterdam, The Netherlands; l Division of Medical Image Computing, Deutsches Krebsforschungszentrum, Heidelberg, Germany; m Pattern Analysis and Learning Group, Department of Radiation Oncology, Heidelberg University Hospital, Heidelberg, Germany; n Departments of Radiology, Urology and Biomedical Data Science, Stanford University, Stanford, CA, USA; o Department of Urinary and Vascular Imaging, Hôpital Edouard Herriot, Hospices Civils de Lyon, Lyon, France; p Faculté de Médecine Lyon-Est, Université Lyon 1, Université de Lyon, Lyon, France; q Department of Urology, Erasmus Medical Center, Rotterdam, The Netherlands; r Department of Radiological Sciences, Oncology and Pathology, Sapienza University of Rome, Rome, Italy; s Division of Surgery and Interventional Sciences, University College London and University College London Hospital, London, UK; t Department of Quantitative Health Sciences, Cleveland Clinic Foundation, Cleveland, OH, USA; u Department of Diagnostic Radiology, Cleveland Clinic Foundation, Cleveland, OH, USA; v Department of Radiology, University Medical Center Groningen, Groningen, The Netherlands; w Department of Radiology, Netherlands Cancer Institute, Amsterdam, The Netherlands; x Department of Circulation and Medical Imaging, Norwegian University of Science and Technology, Trondheim, Norway; y Department of Radiology and Nuclear Medicine, St. Olavs Hospital, Trondheim University Hospital, Trondheim, Norway; z Department of Radiology, Ziekenhuisgroep Twente, Almelo, The Netherland; aa Department of Multi-Modality Medical Imaging, Technical Medical Centre, University of Twente, Enschede, The Netherlands; bb Department of Medical Imaging, Radboud University Medical Center, Nijmegen, The Netherlands

**Keywords:** Prostate cancer, Biparametric magnetic resonance imaging, Multiparametric magnetic resonance imaging, Dynamic contrast-enhanced imaging, Observer study, Noninferiority

## Abstract

**Background and objective:**

Biparametric magnetic resonance imaging (bpMRI), excluding dynamic contrast-enhanced (DCE) magnetic resonance imaging (MRI), is a potential replacement for multiparametric MRI (mpMRI) in diagnosing clinically significant prostate cancer (csPCa). An extensive international multireader multicase observer study was conducted to assess the noninferiority of bpMRI to mpMRI in csPCa diagnosis.

**Methods:**

An observer study was conducted with 400 mpMRI examinations from four European centers, excluding examinations with prior prostate treatment or csPCa (Gleason grade [GG] ≥2) findings. Readers assessed bpMRI and mpMRI sequentially, assigning lesion-specific Prostate Imaging Reporting and Data System (PI-RADS) scores (3–5) and a patient-level suspicion score (0–100). The noninferiority of patient-level bpMRI versus mpMRI csPCa diagnosis was evaluated using the area under the receiver operating curve (AUROC) alongside the sensitivity and specificity at PI-RADS ≥3 with a 5% margin. The secondary outcomes included insignificant prostate cancer (GG1) diagnosis, diagnostic evaluations at alternative risk thresholds, decision curve analyses (DCAs), and subgroup analyses considering reader expertise. Histopathology and ≥3 yr of follow-up were used for the reference standard.

**Key findings and limitations:**

Sixty-two readers (45 centers and 20 countries) participated. The prevalence of csPCa was 33% (133/400); bpMRI and mpMRI showed similar AUROC values of 0.853 (95% confidence interval [CI], 0.819–0.887) and 0.859 (95% CI, 0.826–0.893), respectively, with a noninferior difference of –0.6% (95% CI, –1.2% to 0.1%, *p* < 0.001). At PI-RADS ≥3, bpMRI and mpMRI had sensitivities of 88.6% (95% CI, 84.8–92.3%) and 89.4% (95% CI, 85.8–93.1%), respectively, with a noninferior difference of –0.9% (95% CI, –1.7% to 0.0%, *p* < 0.001), and specificities of 58.6% (95% CI, 52.3–63.1 %) and 57.7% (95% CI, 52.3–63.1%), respectively, with a noninferior difference of 0.9% (95% CI, 0.0–1.8%, *p* < 0.001). At alternative risk thresholds, mpMRI increased sensitivity at the expense of reduced specificity. DCA demonstrated the highest net benefit for an mpMRI pathway in cancer-averse scenarios, whereas a bpMRI pathway showed greater benefit for biopsy-averse scenarios. A subgroup analysis indicated limited additional benefit of DCE MRI for nonexperts. Limitations included that biopsies were conducted based on mpMRI imaging, and reading was performed in a sequential order.

**Conclusions and clinical implications:**

It has been found that bpMRI is noninferior to mpMRI in csPCa diagnosis at AUROC, along with the sensitivity and specificity at PI-RADS ≥3, showing its value in individuals without prior csPCa findings and prostate treatment. Additional randomized prospective studies are required to investigate the generalizability of outcomes.

## 1. Introduction

In men with elevated serum prostate-specific antigen (PSA) levels, magnetic resonance imaging (MRI) has become an important tool in the pathway of prostate cancer (PCa) detection before biopsy [1–6]. To standardize imaging protocols and interpretation of prostate MRI, the Prostate Imaging Reporting and Data System (PI-RADS) was established [7]. Its latest version (PI-RADS version 2.1) recommends a multiparametric MRI (mpMRI) protocol, including T2-weighted (T2W) and diffusion-weighted imaging (DWI), as well as administration of a contrast agent to acquire dynamic contrast-enhanced (DCE) MRI [8]. However, administration of intravenous contrast agents has drawbacks, including cost, time consumption, reduced patient comfort, and potential negative health effects [9]. Meanwhile, DCE MRI has a secondary and limited role in the current reporting system [8].

Given the growing availability and demand of prostate MRI in clinical practice, it is becoming increasingly important to perform prostate MRI examinations more efficiently, cost effectively, and comfortably for patients [10,11]. A noncontrast biparametric MRI (bpMRI) protocol has been shown to achieve comparable diagnostic accuracy for PCa as mpMRI across multiple studies [12–14]. Although shown to be a valid alternative for expert readers, some have reported on the necessity of DCE imaging to assist less experienced readers [15]. Most diagnostic studies have, however, been limited to single-center data and small sample sizes [12–14], raising concerns regarding the potential degradation of bpMRI performance in multi-institutional studies and clinical practice [11,16].

To address these concerns, we performed an extensive international multireader multicase observer study for diagnosing clinically significant PCa (csPCa) using both bpMRI and mpMRI. The primary aim was to assess the noninferior diagnostic performance of bpMRI to that of mpMRI. Additionally, we explored the diagnostic performance of bpMRI and mpMRI at alternative risk thresholds and among both expert and nonexpert groups.

## 2. Patients and methods

The retrospective use of anonymized patient data was approved by review boards at each contributing center (identifiers: REK 2017/576; CMO 2016–3045, project 20011; IRB 2018–597; ZGT23–37) exempting the need to obtain informed consent. The study was conducted following the principles of the Declaration of Helsinki. The Standards for Reporting of Diagnostic Accuracy (STARD) reporting guideline was followed.

### 2.1. Study design

This observer study was part of the Prostate Imaging—Cancer Artificial Intelligence (PI-CAI) challenge, a large confirmatory study with the primary objective of assessing the noninferiority of an artificial intelligence (AI) system in detecting csPCa compared with a panel of radiologists [17]. PI-CAI comprised a large consortium of researchers and a multidisciplinary scientific advisory board with experts across prostate radiology, urology, and AI (Supplementary material). PI-CAI’s study protocol was registered on Clinicaltrial.gov (NCT05489341). Its main outcomes have been detailed elsewhere [17], and its methods are summarized here.

### 2.2. Population

Examinations were obtained between 2015 and 2021 across four European care centers (Radboud University Medical Center, Ziekenhuisgroep Twente, Prostaat Centrum Noord-Nederland, and St. Olav’s Hospital). All examinations were from men with a suspicion of PCa, with elevated serum PSA (≥3 ng/ml) levels and/or abnormal findings on digital rectal examination. Patients with prior prostate treatment or prior csPCa findings were excluded. Extended details on patients included and excluded on a per-center basis have been described elsewhere [17]. Additionally, examinations were assessed for image quality through a two-round review process conducted by key investigators and an expert radiologist (12 yr of experience), excluding examinations with considerable misalignment between sequences and severe artifacts induced by, for example, prostheses, rectal gas, and patient motion (refer to Supplementary Fig. 1 for examples). Image quality–based exclusions were minimized to resemble real-world data closely. From this cohort, a random sample of 400 examinations was included in the observer study. In a post hoc analysis, the image quality of these examinations was assessed by an expert radiologist (refer to the Supplementary material for quality scores and examples of image quality).

### 2.3. Image data

MRI examinations were acquired on 1.5 or 3 Tesla MRI systems from two vendors (Siemens Healthineers, Erlangen, Germany; Philips Medical Systems, Eindhoven, Netherlands) (MRI systems and examination characteristics are detailed in Supplementary Table 1). For all examinations, T2W imaging in three orthogonal planes, axial DWI with high b-value images (b ≥1000 s/mm^2^), apparent diffusion coefficient map, and DCE MRI were obtained.

### 2.4. Histopathology and ground truth definition

Men with PI-RADS 3–5 findings (intermediate, high, and very high risks of csPCa) underwent MRI-targeted biopsy, 12-core systematic biopsies, or both. Men with a negative MRI examination (PI-RADS ≤2) either underwent 12-core systematic biopsies or did not receive any biopsies. In case a patient underwent radical prostatectomy, whole-mount histopathology specimens were obtained. Gleason grade (GG) and Gleason score (GS) were determined according to the International Society of Urological Pathology guidelines [18].

The definition of csPCa was GG ≥2 (GS ≥3 + 4); insignificant PCa (insignPCa) was defined as GG 1 (GS = 3 + 3). All histopathology data were considered to define the reference standard. To confirm the absence of csPCa, at least 3 yr of follow-up data were obtained by a retrospective investigation of institutional records and national registries (eg, the Dutch nationwide PALGA pathology registry). Patient-level outcomes were determined by the highest-graded lesion. Quality control of annotations was executed at each center and verified by researchers at the coordinating center supervised by a radiologist (12 yr of experience).

### 2.5. Observer study

The multireader multicase observer study was conducted via a web-based medical image viewer (grand-challenge.org/reader-studies) and employed a split-plot design to enhance reading efficiency [19,20]. Readers and examinations were allocated randomly into four blocks, stratified by center, csPCa prevalence, and reader experience. All readers were familiar with PI-RADS version 2.1 and reported prostate MRI in clinical practice. Prestudy training was followed to familiarize with the interpretation workflow.

During the study, readers remained blinded to histopathological outcomes and were provided with patient (age, PSA serum level, prostate volume, PSA density [PSAd]) and scanner-specific (vendor and high b-value details) information. Readers sequentially evaluated bpMRI and mpMRI for each patient, following the secondary role in lesion scoring attributed to DCE imaging according to the PI-RADS criteria. Readers allocated PI-RADS 3–5 scores to suspicious lesions and assigned a patient-level suspicion score (ranging from 0 to 100) for the presence of csPCa. After completing the bpMRI assessment, readers were unblinded to the DCE imaging and were allowed to update their findings considering the full mpMRI protocol (Supplementary materials Fig. S2).

Readers were required to assess all examinations within their block and were unable to revisit readings. Following a minimum 5-wk washout period for each reader, a revision round was conducted to address any noncompliant readings (refer to Saha et al. for noncompliant reading examples [17]). During this revision, readers remained blinded to all compliant readings and were granted access only to revisit and correct their noncompliant answers.

### 2.6. Outcome measures

The coprimary outcomes of this study were the patient-level diagnostic performance of csPCa, assessed through the area under the receiver operating characteristic curve (AUROC), alongside the sensitivity and specificity at PI-RADS ≥3. Although the AUROC provides insights into the diagnostic performance across various thresholds, the inclusion of PI-RADS warrants an assessment of the influence of DCE MRI on clinical decision-making [21]. The secondary outcomes included a diagnosis of insignPCa, evaluations of sensitivity and specificity at two alternative risk thresholds—(1) PI-RADS ≥4 along PI-RADS 3 with an elevated PSAd (≥0.15 ng/ml^2^) and (2) PI-RADS ≥4 only, and a subgroup analysis distinguishing between expert (>1000 cases read in total and >200 cases per year) and nonexpert (<1000 cases read in total or <200 cases per year) readers as per the European Society of Urogenital Radiology (ESUR)/European Association of Urology Section of Urologic Imaging (ESUI) criteria [22]. Decision curve analyses (DCAs) incorporating all risk thresholds at bpMRI and mpMRI were performed to investigate the net clinical benefit of each pathway [23,24].

### 2.7. Statistical analysis

The sample size of readers and examinations was determined through an a priori power analysis tailored to address the primary objective of the PI-CAI challenge [17]. Primary outcomes were assessed by binarizing reader results at PI-RADS ≥3, with the highest PI-RADS grade used as the patient-level score, and by constructing empirical receiver operating characteristic curves based on patient-level suspicion scores. A multireader multicase analysis of variance using the Obuchowski-Rockette [25] method was used to derive the mean estimates and corresponding 95% Wald confidence intervals (CIs) for the empirical AUROC, as well as the sensitivities and specificities at PI-RADS ≥3. For an explorative secondary analysis, reader results were binarized at alternative risk thresholds, and readers were divided into expert and nonexpert groups. DCAs were assessed across threshold probabilities ranging from 5% to 30% [26]. These probabilities represent the willingness to perform biopsies to diagnose csPCa. For instance, a threshold of 10% indicates one csPCa found per ten biopsies performed. The net benefit was calculated as the true positive count corrected for the false positive count weighted by the odds of a threshold probability, divided by the cohort size [23,24].

Statistical tests were reserved for the primary outcomes [27] and were conducted according to a preapproved statistical analysis plan (Supplementary material). Noninferiority tests comparing bpMRI with mpMRI, using AUROC, and sensitivity and specificity at PI-RADS ≥3, were conducted with a 5% noninferiority margin and a base-level significance threshold of *p* < 0.05 [28]. To control the family-wise error rate, a prespecified hierarchical tree and a Holm-Bonferroni correction were implemented. All statistical analyses were performed with R version 2022.12.0 software and the MRMCaov package (R Foundation for Statistical Computing, Vienna, Austria) [29,30].

## 3. Results

### 3.1. Patient and reader characteristics

The prevalence of csPCa was 33% (133/400). Table 1 presents the patient and examination characteristics per center, PI-RADS scores per clinical routine, and GGs (for lesion level characteristics per center and population characteristics per reader block, refer to Supplementary Tables 2 and 3).

Assessments were conducted by 62 readers from 45 centers spanning 20 countries. Following the ESUR/ESUI criteria, 46 (74%) readers were classified as experts and 16 (26%) as non-experts [17]. The analysis was performed on 6174 reads (99.6% of all interpretations) after excluding 26 patient assessments (<1%) due to unsolved issues (STARD diagram of the observer study available in Saha et al. [17]).

### 3.2. Diagnostic performances

**Fig. 1 –:**
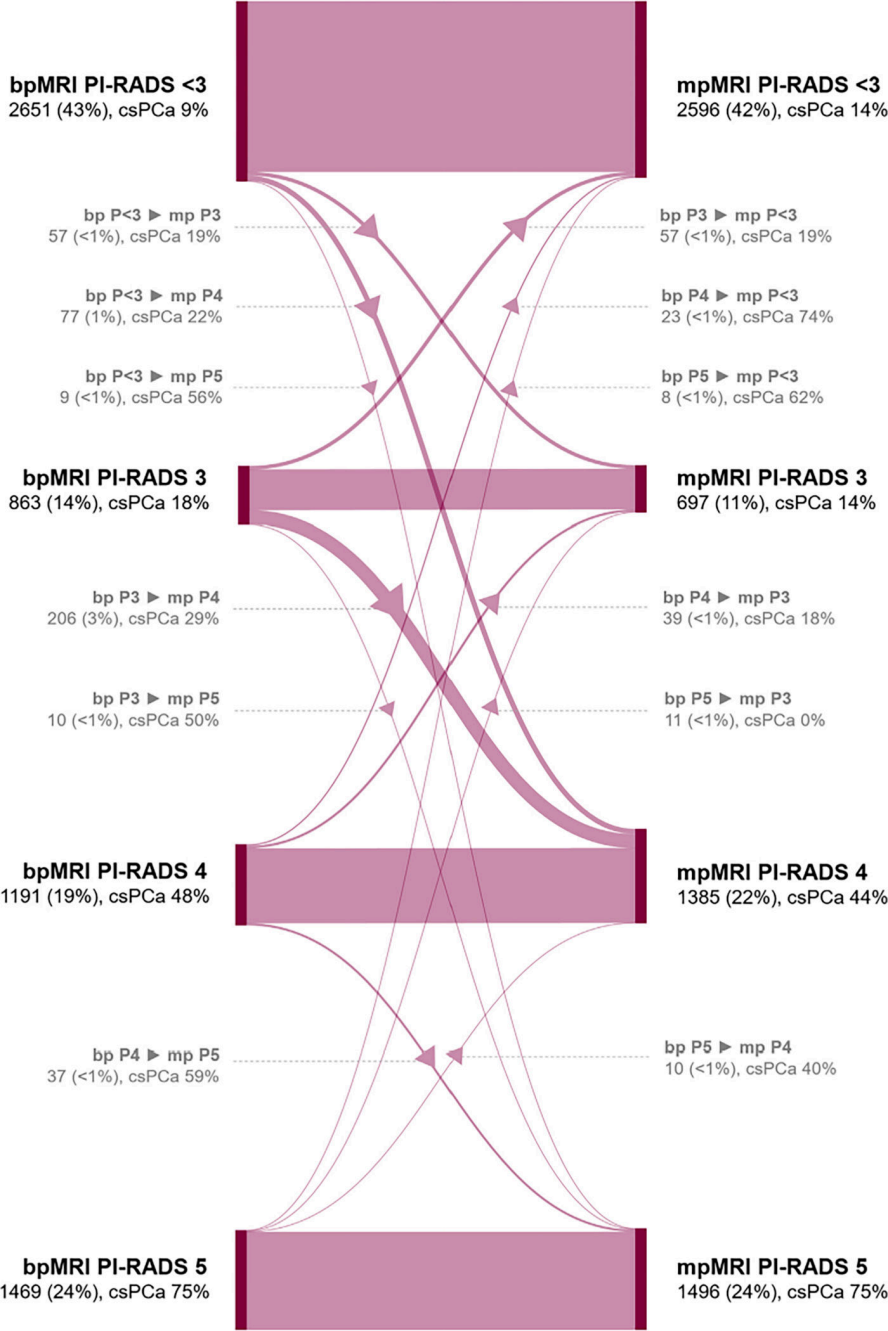
Flow diagram illustrating patient-level PI-RADS scores at biparametric (bp) and multiparametric (mp) MRI, encompassing all readings within the observer study (*n* = 6174). The diagram depicts intrareader consistency, and upgrades or downgrades between bpMRI and mpMRI assessments. The left side represents bpMRI readings, while the right side represents mpMRI readings. Arrows indicate upgrades and downgrades from bpMRI to mpMRI. Each category includes the number of readings, percentages, and its clinically significant prostate cancer (csPCa) prevalence. For 91% of readings (*n* = 5630), bpMRI and mpMRI yielded the same patient-level PI-RADS score. A minority of readings involved patients initially classified with a PI-RADS <3 score at bpMRI, and subsequently upgraded to PI-RADS 3 (*n* = 57 [<1%], with 19% csPCa), PI-RADS 4 (*n* = 77 [1%], with 22% csPCa), and PI-RADS 5 (*n* = 9 [<1%], with 56% csPCa) with mpMRI. Another significant group comprised readings initially assigned an equivocal grade (PI-RADS = 3) but were subsequently upgraded to a PI-RADS score of ≥4 (*n* = 206 [3%], with 29% csPCa) and 5 (*n* = 10 [<1%], with 50% csPCa). At mpMRI, there was an overall decrease in equivocal PI-RADS = 3 scores of 3% (from 14% to 11%). MRI = magnetic resonance imaging; P/PI-RADS = Prostate Imaging Reporting and Data System.

It was observed that bpMRI had noninferior diagnostic performance to mpMRI in AUROC (difference of –0.6% [95% CI, –1.2% to 0.1%], *p* < 0.001), and sensitivity (difference of –0.9% [95% CI, –1.7% to 0.0%], *p* < 0.001) and specificity (difference of 0.9% [95% CI, 0.0–1.8%], *p* < 0.001) at PI-RADS ≥3 (refer to Tables 2 and 3 for diagnostic performances). Per-reader differences for the primary endpoints are available in Supplementary Figure 3. A similar number of csPCa diagnoses were identified with bpMRI (117/133 [interquartile range {IQR}: 112–124]) to those identified with mpMRI (118/133 [IQR: 112–124]), as well as a comparable number of insignPCa diagnoses (39 [IQR: 32–44] vs 40 [IQR: 32–44]). Figure 1 presents a flow diagram of the patient-level PI-RADS scores at bpMRI and mpMRI. For 91% of readings (5630/6174), bpMRI and mpMRI had the same patient-level PI-RADS score (refer to Supplementary Table 4 for the zonal distribution of patient-level PI-RADS scores at bpMRI and mpMRI).

### 3.3. Alternative risk thresholds

Compared with bpMRI, mpMRI was associated with increased sensitivity at the cost of higher false-positive diagnoses at both alternative risk thresholds (see Table 3). At a threshold including examinations with PI-RADS ≥4 and PI-RADS 3 for men with PSAd ≥0.15 ng/ml^2^, bpMRI exhibited 1.6% (95% CI, 0.7–2.4%) lower sensitivity and 2.9% (95% CI, 1.7–4.1%) higher specificity than mpMRI. The tradeoff between sensitivity and specificity was greater at PI-RADS ≥4, with bpMRI exhibiting 3.8% (95% CI, 2.3–5.2%) lower sensitivity and increased specificity of 3.5% (95% CI, 2.2–4.8%).

### 3.4. Decision curve analyses

**Fig. 2 –:**
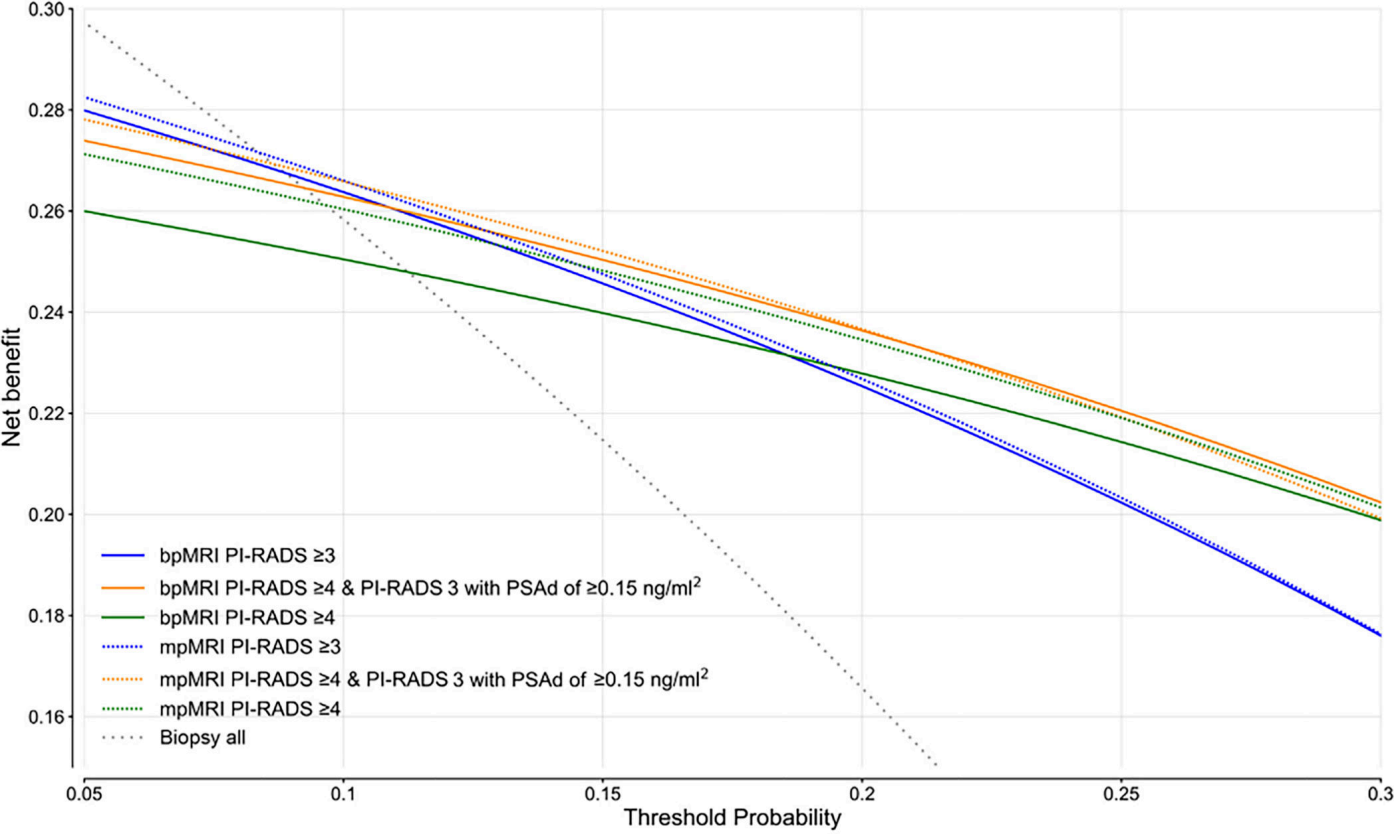
Decision curve analysis for diagnosing clinically significant prostate cancer (csPCa) with biparametric (bp) and multiparametric (mp) MRI across three different risk thresholds. Threshold probabilities range from 5% to 30%, representing the willingness to conduct biopsies to diagnose one case of csPCa. Lower thresholds represent cancer-averse scenarios, while higher thresholds indicate biopsy-averse scenarios. The net benefit (NB) is defined as the true positive count corrected for false positives, weighted by the odds of the threshold probability. Across thresholds, mpMRI at PI-RADS ≥3, mpMRI at PI-RADS ≥4 and PI-RADS 3 with prostate-specific antigen density (PSAd) of ≥0.15 ng/ml^2^, as well as bpMRI at PI-RADS ≥4 and PI-RADS 3 with PSAd of ≥0.15 ng/ml^2^ exhibited the highest NB. Differences in NB between pathways were small. At an European Association of Urology–recommended 10% probability threshold [5], the NB difference between bpMRI and mpMRI at PI-RADS ≥3 was 0.002, indicating that 1/0.002 = 500 mpMRI examinations are needed to diagnose one additional csPCa case compared with a bpMRI protocol. MRI = magnetic resonance imaging; PI-RADS = Prostate Imaging Reporting and Data System.

Figure 2 shows DCAs across all pathways, incorporating bpMRI and mpMRI across all risk thresholds. With threshold probabilities ranging from 5% to 10%, reflecting cancer-averse scenarios and adhering to recommended biopsy thresholds defined by the European Association of Urology guidelines [5], mpMRI achieved the highest net benefit by restricting biopsies to PI-RADS ≥3 examinations. Between 10% and 22%, the highest net benefit was observed for mpMRI with PI-RADS ≥4 and PI-RADS 3 for men with a PSAd ≥0.15 ng/ml^2^ threshold. Between 22% and 30% thresholds, bpMRI limiting biopsies to PI-RADS ≥4 and PI-RADS 3 for men with PSAd ≥0.15 ng/ml^2^ examinations demonstrated the highest net benefit. A DCA with the net benefit expressed as the net reduction in interventions is shown in Supplementary Figure 4.

### 3.5. Expert versus nonexpert readers

The diagnostic performances of expert and nonexpert readers are shown in Tables 2 and 3. Overall, expert readers achieved higher diagnostic performances than nonexperts (AUROC of 0.863 [95% CI, 0.829–0.897] vs 0.824 [95% CI, 0.784–0.864] at bpMRI, and AUROC of 0.869 [95% CI, 0.835–0.903] vs 0.833 [95% CI, 0.792–0.873] at mpMRI). Only a limited additional value of DCE MRI was observed for nonexperts compared with experts (pairwise difference of 0.6% [95% CI, –1.2 to 0.0] for experts and 0.9% [95% CI, –1.7 to 0.0] for nonexperts). Similar trends were observed across other test metrics and risk thresholds.

## 4. Discussion

The PI-CAI reader study showed that bpMRI is noninferior to mpMRI in the diagnosis of csPCa, based on AUROC, and sensitivity and specificity at PI-RADS ≥3 in men without prior csPCa findings and prostate treatment. At alternative risk thresholds, mpMRI was associated with higher sensitivities at the cost of decreased specificities. DCAs showed that two mpMRI pathways had the highest net benefit below a 22% threshold probability, with a bpMRI pathway being favorable above this threshold. Finally, we did not observe substantial additional benefit from using DCE MRI for non-expert readers.

Our results support current lower-level evidence indicating similar csPCa diagnostic performance between bpMRI and mpMRI. In a systematic review from Bass et al [14] pooling the results of 44 studies, bpMRI was associated with an AUROC of 0.870. Within a head-to-head comparison of bpMRI and mpMRI, including a subset of 17 studies, the authors showed no significant difference in sensitivity and specificity between the two protocols, with sensitivities of 84% (95% CI, 73–91%) and 89% (95% CI, 80–94%), and specificities of 79% (95% CI, 70–85%) and 74% (95% CI, 56–87%).

In the context of an MRI-directed biopsy pathway, the PI-RADS score is important in patient selection for biopsy [21]. We found that the majority of readings showed consistent PI-RADS scores between bpMRI and mpMRI, with a small fraction changing from PI-RADS <3 to PI-RADS ≥3. These findings demonstrate the high diagnostic value of bpMRI, especially at a low risk threshold, minimizing the risk of missing aggressive cancer. In line with the role of DCE MRI as per PI-RADS version 2.1, DCE MRI helped reduce the number of intermediate scores, which might benefit the clinical management of these patients. Consistent with our findings, Zawaideh et al [31] reported high similarity between bpMRI and mpMRI PI-RADS scores (86% of patients) and an 8.7% reduction in PI-RADS 3 cases. Similarly, van der Leest et al [32] and El-Shater Bosaily et al [33] reported reductions in equivocal scores of 1.4% and 4.4%, respectively. Additionally, we observed a 3% (206/6174 readings) incidence of upgrades from PI-RADS 3 to 4, with 29% of these upgrades exhibiting csPCa. A recent prospective study similarly observed a high number of false positives within this group [34].

The DCAs showed that in cancer-averse scenarios, mpMRI remains favorable, achieving a net benefit exceeding that of bpMRI strategies. This aligns with the findings of de Oliveira Correia et al [26], highlighting a substantial positive impact on biopsy decisions when using PI-RADS upgrading rules. The European Association of Urology recommends biopsies at a 5–10% risk threshold [6]. The net benefit difference at a 10% threshold implies the requirement for 500 mpMRI examinations to diagnose one additional csPCa case, prompting deliberation on whether it is acceptable to subject a large number of patients to DCE imaging. At the willingness to perform fewer than five biopsies per csPCa case diagnosed (>20% probability threshold), we observed that bpMRI with PI-RADS ≥4 and PI-RADS 3 for men with an elevated PSAd outperformed the mpMRI pathway, consistent with the findings of Van der Leest et al [32].

Although nonexpert readers had overall lower diagnostic performance, only a limited additional value of using DCE MRI compared with expert readers was observed, contradicting previous findings [15]. In the work by Gatti et al [15], the nonexpert reader group included two junior radiologists and two residents, while the nonexpert group in this study was larger and comprised radiologists with varying levels of experience. Our decision to adhere to the expertise criteria as per the ESUR/ESUI consensus statements [22] aimed to standardize expertise groups and ensure sufficient sample sizes for an analysis. The disparities in the study cohorts, reader expertise, and group sizes may have contributed to the observed differences.

Our study exhibits several strengths. First, diagnostic performance was evaluated at both AUROC and PI-RADS thresholds, allowing for the evaluation of the added value of DCE MRI within and beyond PI-RADS standards [35]. Second, the inclusion of a large sample size of readers and examinations from diverse centers across multiple countries ensures comprehensive heterogeneity. Additionally, the determination of the presence or absence of csPCa was based on all available histopathological evidence and a follow-up period of ≥3 yr. This minimizes limitations such as biased whole-mount histopathology cohorts and reliance solely on biopsy outcomes, which might be prone to sampling errors.

This study is not without limitations. First, the decision to acquire biopsies in clinical routine was based on mpMRI, which introduces a verification bias in our reference standard. Second, the reading of bpMRI and mpMRI followed a sequential rather than a crossover design, potentially introducing a sequential reading bias. Nonetheless, this aligns with clinical practice, where T2W imaging and DWI play a primary role in the interpretation, with DCE MRI serving as a complementary tool [8]. Third, examinations involving previous prostate treatment and severe image artifacts were excluded, which may underestimate the value of DCE imaging in such scenarios. Despite our exclusion criteria, a wide range of image quality was still observed in our study (see the Supplementary material). Including only examinations with optimal image quality would considerably reduce the representation of the real-world data [36]. Nevertheless, high-quality bpMRI acquisition is crucial, as DCE imaging cannot compensate for low-quality T2W and DWI sequences in this setting, and should therefore be a prerequisite for safe clinical implementation [37]. Fourth, a majority of our reader population comprised expert readers, and the majority of the studies included were obtained from high-throughput centers. Lastly, csPCa was defined as GG ≥2, whereas some studies have used different definitions [38–40].

To bridge the gap between current limitations and future research, upcoming work should focus on multicenter prospective studies to validate the transferability of evidence for bpMRI to new patients, as outlined in emerging initiatives [41,42]. Additionally, it is recommended to evaluate the PI-RADS version 2.1 scoring system, which was originally developed for mpMRI, to evaluate whether accurate scoring of bpMRI, as well as its overall diagnostic performance, can be enhanced further through, for instance, the integration of quantitative MRI [43] and AI assistance [16,17].

## 5. Conclusions

Our findings demonstrate that the diagnostic capability of bpMRI in detecting csPCa was noninferior to that of mpMRI in men without prior csPCa findings and prostate treatment, as determined by the AUROC, as well as the sensitivity and specificity at PI-RADS ≥3. While the addition of DCE MRI at elevated risk thresholds resulted in higher sensitivities, it came at the expense of increased false positive diagnoses. Notably, a limited additional advantage for DCE MRI was observed for nonexpert readers. Consequently, bpMRI emerges as a promising alternative to mpMRI, offering a potential solution to the increasing demand for prostate MRI examinations. Prospective evaluation is necessary to confirm this large-scale retrospective evidence to ensure safe clinical implementation.

## Supplementary Material

Supplementary Material

## Figures and Tables

**Table 1 –:** Patient distribution and characteristics of the observer study

Data	RUMC, Nijmegen	ZGT, Twente	PCNN, Groningen	STOH, Trondheim	Total
No. of sites	2	1	8	1	12
No. of MRI scanners	2 S	1 S	2 S, 1 P	1 S	3 S, 1 P
No. of patients	135	106	79	80	400
Age (yr), median (IQR)	65 (59–69)	65 (58–68)	66 (63–72)	67 (61–70)	66 (60–69)
PSA (ng/ml), median (IQR)	6.9 (5.1–9.6)	6.4 (5.2–8.6)	9.6 (6.8–15)	6.9 (5.4–10.2)	7.1 (5.4–10.2)
Prostate volume (ml), median (IQR)	61 (45–84)	51 (40–76)	47 (35–64)	50 (35–72)	54 (40–77)
Median PSAd (ng/ml^2^) (IQR)	0.11 (0.08–0.16)	0.12 (0.08–0.18)	0.18 (0.13–0.29)	0.13 (0.08–0.20)	0.13 (0.09–0.21)
No. of benign or insignPCa cases (%)	96 (71)	73 (69)	46 (58)	52 (65)	267 (66)
No. of csPCa cases	39 (29)	33 (31)	33 (42)	28 (35)	133 (34)
With index lesion in PZ	35 (89)	30 (90)	27 (82)	21 (75)	113 (85)
With index lesion in TZ ^a^	4 (11)	3 (10)	6 (18)	7 (25)	20 (15)
No. of reference for patient (%)					
No hist. with follow-up ^b^	55 (41)	0 (0)	0 (0)	17 (21)	72 (18)
Sys. TRUSBx	24 (18)	51 (48)	24 (30)	39 (49)	138 (35)
MRGBx	9 (7)	0 (0)	43 (54)	0 (0)	52 (13)
MRGBx + Sys. TRUS	37 (27)	32 (30)	0 (0)	9 (11)	78 (20)
Radical prostatectomy	10 (7)	23 (21)	12 (15)	15 (19)	60 (15)
No. of GG scores (%) ^c^					
GG1	16 (12)	23 (22)	16 (20)	3 (4)	58 (15)
GG2	20 (15)	23 (22)	15 (19)	9 (11)	67 (18)
GG3	10 (7)	4 (4)	13 (16)	11 (14)	38 (10)
GG4	2 (1)	2 (2)	2 (3)	4 (5)	10 (3)
GG5	7 (5)	4 (4)	3 (4)	4 (5)	18 (5)
No. of PI-RADS scores (%) ^c,d^					
PI-RADS 1–2	82 (61)	52 (49)	16 (20)	44 (55)	194 (49)
PI-RADS 3	5 (4)	6 (6)	9 (11)	8 (10)	28 (7)
PI-RADS 4	17 (13)	18 (17)	28 (35)	7 (9)	70 (18)
PI-RADS 5	31 (23)	30 (28)	26 (33)	21 (26)	108 (27)

csPCa = clinically significant prostate cancer (GG ≥2); GG = Gleason grade; hist. = histopathology; insignPCa = clinically insignificant prostate cancer (GG = 1); IQR = interquartile range; MRI = magnetic resonance imaging; MRGBx = MRI guided biopsy; No. = number; P = Phillips Medical Systems MRI scanner; PCNN = Prostaat Centrum Noord-Nederland, the Netherlands; PI-RADS = Prostate Imaging Reporting and Data System; PSA = prostate-specific antigen; PSAd = prostate-specific antigen density; PZ = peripheral zone; RUMC = Radboud University Medical Center, the Netherlands; S = Siemens Healthineers MRI scanner; STOH = St. Olav’s Hospital, Trondheim University Hospital, Norway; Sys. TRUSBx = systematic transrectal ultrasound guided biopsy; TRUS = transrectal ultrasound; TZ = transition zone; ZGT = Ziekenhuisgroep Twente, the Netherlands.

a Includes lesions (partly) located in the central zone and anterior fibromuscular stroma.

b Follow-up period of at least 3 yr.

c Defined as the highest score found on a per-patient level.

d As determined by the original radiologist report from clinical routine.

**Table 2 –:** Area under the ROC curve (AUROC) performances

	AUROC bpMRI (95% CI)	AUROC mpMRI (95% CI)	Pairwise difference (bpMRI – mpMRI) % (95% CI)
All readers (*n* = 62)	0.853 (0.819–0.887)	0.859 (0.826–0.893)	−0.6 (−1.2 to 0.1)
Expert readers (*n* = 46)	0.863 (0.829–0.897)	0.869 (0.835–0.903)	−0.6 (−1.2 to 0.0)
Nonexpert readers (*n* = 16)	0.824 (0.784–0.864)	0.833 (0.792–0.873)	−0.9 (−1.7 to 0.0)

AUROC = area under the ROC curve; bpMRI = biparametric MRI; CI = confidence interval; mpMRI = multiparametric MRI; MRI = magnetic resonance imaging;

**Table 3 –:** Diagnostic performances and differences of bpMRI and mpMRI at operating points

	bpMRI		mpMRI		Pairwise differences (bpMRI – mpMRI)	
	Sens	Spec	Sens	Spec	Sens	Spec
	% (95% CI)	% (95% CI)	% (95% CI)	% (95% CI)	% (95% CI)	% (95% CI)
PI-RADS ≥3						
All readers ^a^	88.6 (84.8–92.3)	58.6 (53.2–64.0)	89.4 (85.8–93.1)	57.7 (52.3–63.1)	−0.9 (−1.7 to 0.0)	0.9 (0.0–1.8)
Expert readers ^b^	89.7 (85.9–93.4)	58.3 (52.0–64.6)	90.2 (86.4–93.9)	57.6 (51.3–63.9)	−0.5 (−1.3 to 0.3)	0.7 (0.4–1.7)
Nonexpert readers ^c^	85.4 (80.3–90.6)	59.5 (51.8–67.1)	87.3 (83.1–91.5)	58.0 (50.0–66.0)	−1.9 (−4.1 to 0.3)	1.5 (0.0–3.0)
PI-RADS ≥4 or PI-RADS 3 with PSAd ≥0.15						
All readers ^a^	85.4 (80.8–90.0)	71.5 (66.6–76.4)	87.0 (82.5–91.4)	68.6 (63.7–73.4)	−1.6 (−2.4 to −0.7)	2.9 (1.7–4.1)
Expert readers ^b^	86.4 (81.7–91.1)	71.0 (65.4–76.7)	87.8 (83.3–92.3)	68.1 (62.5–73.7)	−1.4 (−2.4 to −0.4)	2.9 (1.5–4.3)
Nonexpert readers ^c^	82.6 (77.1–88.0)	72.8 (66.5–79.2)	84.7 (79.5–89.8)	69.9 (63.3–76.5)	−2.1 (−3.4 to 0.8)	2.9 (1.0–4.9)
PI-RADS ≥4						
All readers ^a^	80.8 (75.6–85.9)	75.6 (71.0–80.3)	84.5 (79.7–89.3)	72.1 (67.5–76.7)	−3.8 (−5.2 to −2.3)	3.5 (2.2–4.8)
Expert readers ^b^	81.5 (76.2–86.9)	75.4 (70.1–80.7)	85.1 (80.2–90.1)	71.8 (66.5–77.1)	−3.6 (−5.1 to −2.2)	3.6 (2.1–5.1)
Non-expert readers ^c^	78.6 (71.9–85.4)	76.3 (69.8–82.7)	82.8 (76.8–88.7)	73.1 (66.6–79.7)	−4.1 (−7.2 to −1.1)	3.1 (1.0–5.2)

bpMRI = biparametric MRI; CI = confidence interval; mpMRI = multiparametric MRI; MRI = magnetic resonance imaging; PI-RADS = Prostate Imaging Reporting and Data System; PSAd = prostate-specific antigen density; Sens = sensitivity; Spec = specificity.

a Number of readers = 62.

b Number of readers = 46.

c Number of readers = 16.

## ADVANCING PRACTICE

### What does this study add?

We conducted a large-scale international multireader study involving 62 radiologists worldwide, utilizing a large dataset comprising 400 multicenter prostate magnetic resonance imaging (MRI) examinations. This approach addresses the limitations of previous studies, which often relied on comparative diagnostic accuracy studies conducted with single-center data and smaller reader populations, potentially limiting the generalizability of their findings. Our results demonstrate that biparametric MRI (bpMRI) is noninferior to multiparametric MRI (mpMRI) in diagnosing clinically significant prostate cancer, as assessed by the area under the receiver operating curve, and the sensitivity and specificity at a Prostate Imaging Reporting and Data System threshold of ≥3. Through a secondary analysis, we highlight that less experienced readers gain limited additional advantage from dynamic contrast-enhanced MRI, whereas in scenarios prioritizing cancer avoidance, mpMRI offers incrementally higher net benefit than bpMRI. Our study adds clear, definitive, and large-scale retrospective evidence supporting the diagnostic accuracy of bpMRI, and will support the outcomes of upcoming prospective clinical trials investigating similar objectives.

### Clinical Relevance

This is a well done retrospective study at several high volume centers showing non-inferiority of bi-parametric compared to multiparametric MRI. While these findings necessitate prospective evaluation, the result of demonstrated non-inferiority would result in shorter study time, removing the need for physician presence for contrast administration thereby increasing patient throughput and therefore access to prostate MRI. The upcoming prospective comparison in the PRIME study (ref 37019486) will provide further insight into the validity of this approach for selected patients. Associate Editor Brian Francis Chapin, M.D.

### Patient Summary

In this study, we investigated whether a shorter magnetic resonance imaging (MRI) protocol, without the use of a contrast agent, was not inferior in diagnosing clinically significant prostate cancer as a protocol that includes contrast. We analyzed the assessments of 62 radiologists on 400 prostate MRI examinations and found that the noncontrast protocol demonstrated similar capability in diagnosing clinically significant prostate cancer in a patient population without prior prostate treatment and previously identified clinically significant cancer. We suggest prospective studies where biopsy decisions are made with and without a contrast agent to see whether these promising results hold up in clinical practice.
