# Supplementary material for: Evaluating Biparametric Versus Multiparametric Magnetic Resonance Imaging for Diagnosing Clinically Significant Prostate Cancer: An International, Paired, Noninferiority, Confirmatory Observer Study
Source: Eur Urol. Author manuscript; Available in PMC 2025 Feb 1. (PMC11769734; doi:10.1016/j.eururo.2024.09.035)
Supplement: Supplementary Material [file NIHMS2033270-supplement-Supplementary_Material.docx]

**Supplementary material**

**Section S1. List of PI-CAI consortium members** *(part of the observer study)*

**Section S2. Post-hoc image quality assessment**

**Section S3. Statistical Analysis Plan** *(including power analysis of PI-CAI)*

**Section S4. Supplementary Figures**

Supplementary Figure 1. Examples of excluded examinations due to insufficient image quality

Supplementary Figure 2. Example of the observer study workflow environment

Supplementary Figure 3. Per reader differences in primary diagnostic outcomes

Supplementary Figure 4. Decision Curve Analysis, number of interventions avoided

**Section S4. Supplementary Tables**

Supplementary Table 1. MRI protocols

Supplementary Table 2. Lesion-level characteristics of the observer study

Supplementary Table 3. Patient distribution and characteristics of the observer study per split-plot

Supplementary Table 4. Zonal distribution of patient-level PI-RADS scores at bpMRI and mpMRI

**Section S1. List of PI-CAI consortium members** *(part of the observer study)*

**Investigators**

Jasper J. Twilt, M.Sc., Radboud University Medical Center, The Netherlands

Anindo Saha, M.Sc., Radboud University Medical Center, The Netherlands

Joeran S. Bosma, M.Sc., Radboud University Medical Center, The Netherlands

Bram van Ginneken, Ph.D., Radboud University Medical Center, The Netherlands

Constant R. Noordman, M.Sc., Radboud University Medical Center, The Netherlands

Ivan Slootweg, B.Sc., Radboud University Medical Center, The Netherlands

Christian Roest, M.Sc., University Medical Center Groningen, The Netherlands

Stefan J. Fransen, M.Sc., University Medical Center Groningen, The Netherlands

Mohammed R. S. Sunoqrot, Ph.D., Norwegian University of Science and Technology, St. Olavs Hospital, Trondheim University Hospital; Norway

Tone F. Bathen, Ph.D., Norwegian University of Science and Technology, St. Olavs Hospital, Trondheim University Hospital; Norway

Dennis Rouw, M.D., Martini Hospital Groningen, The Netherlands

Jeroen Geerdink, B.Sc., Ziekenhuis Groep Twente, The Netherlands

Chris van Run, M.Sc., Radboud University Medical Center, The Netherlands

Miriam Groeneveld, M.Sc., Radboud University Medical Center, The Netherlands

James Meakin, Ph.D., Radboud University Medical Center, The Netherlands

Jos J.F.M. Immerzeel, M.D., Andros Clinics, The Netherlands

Derya Yakar, M.D., Netherlands Cancer Institute and University Medical Center Groningen, The Netherlands

Mattijs Elschot, Ph.D., Norwegian University of Science and Technology, St. Olavs Hospital, Trondheim University Hospital, Norway

Jeroen Veltman, M.D., Ziekenhuis Groep Twente and University of Twente, The Netherlands

Jurgen J. Fütterer, M.D., Radboud University Medical Center, The Netherlands

Maarten de Rooij, M.D., Radboud University Medical Center, The Netherlands

Henkjan Huisman, Ph.D., Radboud University Medical Center, The Netherlands; Norwegian University of Science and Technology, Norway

**Scientific Advisory Board (SAB)**

Anders Bjartell, M.D., Skåne University Hospital and Lund University Cancer Centre, Sweden

Anwar R. Padhani, M.D., Mount Vernon Cancer Centre, United Kingdom

David Bonekamp, M.D., Deutsches Krebsforschungszentrum Heidelberg, Germany

Geert Villeirs, M.D., Ghent University Hospital, Belgium

Georg Salomon, M.D., University Hospital Hamburg-Eppendorf, Germany

Gianluca Giannarini, M.D., Santa Maria della Misericordia University Hospital, Italy

Henkjan Huisman, Ph.D., Radboud University Medical Center, The Netherlands; Norwegian University of Science and Technology, Norway

Jayashree Kalpathy-Cramer, Ph.D., University of Colorado, United States

Jelle Barentsz, M.D., Andros Clinics, The Netherlands

Klaus H. Maier-Hein, Ph.D., Heidelberg University Hospital and Deutsches Krebsforschungszentrum Heidelberg, Germany

Mattijs Elschot, Ph.D., Norwegian University of Science and Technology, St. Olavs Hospital, Trondheim University Hospital; Norway

Mirabela Rusu, Ph.D., Stanford University, United States

Nancy A. Obuchowski, Ph.D., Cleveland Clinic Foundation, United States

Olivier Rouviere, M.D., Hospices Civils de Lyon, France

Roderick van den Bergh, M.D., Erasmus Medical Center, The Netherlands

Valeria Panebianco, M.D., Sapienza University of Rome, Italy

Veeru Kasivisvanathan, M.D., University College London and University College London Hospital, United Kingdom

**Radiologists in the Observer Study**

Afsoun Malakoti-Fard, M.D., Denmark University Hospital Herlev, Denmark

Agnė Mačiūnien, M.D., Republican Klaipeda Hospital, Lithuania

Akira Kawashima, M.D., Mayo Clinic Arizona, United States

Ana M. Gaivão, M.D., Champalimaud Foundation, Portugal

Ana S. L. Moreira, M.D., Centro Hospitalar Universitário do Algarve, Portugal

Andrea Ponsiglione, M.D., University of Naples Federico II, Italy

Annelies Rappaport, M.D., Sint Trudo Hospital, Belgium

Arnaldo Stanzione, M.D., University of Naples Federico II, Italy

Arturas Ciuvasovas, M.D., Republican Klaipeda Hospital, Lithuania

Baris Turkbey, M.D., National Cancer Institute, National Institutes of Health, United States

Bart De Keyzer, M.D., Sint Trudo Hospital, Belgium

Bodil G. Pedersen, M.D., Aarhus University Hospital, Denmark

Bram Eijlers, M.D., Erasmus University Medical Center, The Netherlands

**Section S1. List of PI-CAI consortium members** *(part of the observer study) (continued)*

Christine Chen, M.D., Mount Sinai Health System, United States

Ciabattoni Riccardo, M.D., Ospedale San Salvatore di Pesaro, Azienda Sanitaria Territoriale Pesaro-Urbino, Italy

Deniz Alis, M.D., Acibadem Mehmet Ali Aydinlar University School of Medicine, Turkey

Ewout F.W. Courrech Staal, M.D., Maasstad Hospital, The Netherlands

Erik Thimansson, M.D., Helsingborg Hospital, Sweden

Fredrik Jäderling, M.D., Capio Saint Göran’s Hospital, Karolinska Institutet, Sweden

Fredrik Langkilde, M.D., Sahlgrenska University Hospital, Sweden

Giacomo Aringhieri, M.D., University of Pisa, Italy

Giorgio Brembilla, M.D., IRCCS Ospedale San Raffaele, Italy

Hannah Son, M.D., Sydney Adventist Hospital, Australia

Hans van der Lelij, M.D., Maasstad Ziekenhuis, The Netherlands

Henricus P. J. Raat, M.D., Laurentius Hospital Roermond, The Netherlands

Ingrida Pikūnienė, M.D., Affidea, Lithuania

Iva Macova, M.D., First Faculty of Medicine, Charles University, Czech Republic

Ivo Schoots, M.D., Erasmus University Medical Center, Netherlands Cancer Institute, The Netherlands

Iztok Caglic, M.D., University of Cambridge and Cambridge University Hospitals, United Kingdom

Jeries P. Zawaideh, M.D., IRCCS Ospedale Policlinico San Martino, Italy

Jonas Wallström, M.D., Sahlgrenska University Hospital, Sweden

Leonardo K. Bittencourt, M.D., University Hospitals & Case Western Reserve University, United States

Misbah Khurram, M.D., Herlev and Gentofte Hospital Copenhagen, Denmark

Moon Hyung Choi, M.D., Eunpyeong St. Mary’s Hospital, The Catholic University of Korea, South Korea

Naoki Takahashi, M.D., Mayo Clinic Minnesota, United States

Nelly Tan, M.D., Mayo Clinic Arizona, United States

Olivier Rouviere, M.D., Hospices Civils de Lyon, France

Paolo N. Franco, M.D., Fondazione IRCCS San Gerardo dei Tintori, Italy

Patricia A. Gutierrez, M.D., CHU Lille, France

Petr Hanus, M.D., First Faculty of Medicine, Charles University, Czech Republic

Philippe Puech, M.D., CHU Lille, France

Philipp R. Rau, M.D., CINOV, Switzerland

Pieter de Visschere, M.D., Ghent University Hospital, Belgium

Ramette Guillaume, M.D., CH Valenciennes, France

Renato Cuocolo, M.D., University of Salerno, Italy

Ricardo O. Falcão, M.D., Grupo Alliança, Brazil

Rogier S. A. van Stiphout, M.D., Laurentius Hospital Roermond, The Netherlands

Rossano Girometti, M.D., University of Udine and University Hospital S. Maria della Misericordia (ASUFC), Italy

Ruta Briediene, M.D., Affidea, National Cancer Institute Lithuania, Lithuania

Rūta Grigienė, M.D., Affidea, National Cancer Institute Lithuania, Lithuania

Samuel Gitau, M.D., Aga Khan University Hospital, Kenya

Samuel Withey, M.D., Royal Marsden Hospital, United Kingdom

Sangeet Ghai, M.D., University Health Network, University of Toronto, Canada

Tobias Penzkofer, M.D., Charité-Universitätsmedizin Berlin, Germany

Tristan Barrett, M.D., University of Cambridge, United Kingdom

Valeria Panebianco, M.D., Sapienza University of Rome, Italy

Varaha Sai Tammisetti, M.D., University of Texas McGovern Medical School, United States

Vibeke B. Løgager, M.D., Denmark University Hospital Herlev, Denmark

Vladimír Černý, M.D., First Faculty of Medicine, Charles University, Czech Republic

Wulphert Venderink, M.D., Radboud University Medical Center, The Netherlands

Yan Mee Law, M.D., Singapore General Hospital, Singapore

Young Joon Lee, M.D., Eunpyeong St. Mary’s Hospital and The Catholic University of Korea, South Korea

**Section S2. Post-hoc image quality assessment**

In a post-hoc analysis, all 400 examinations were assessed by an expert radiologist (12 years of experience in reading prostate MRI) for image quality using the updated prostate imaging quality scoring system (PI-QUALv2) [1].

Section S2 Table 1 details the overall PI-QUAL scores for bpMRI and mpMRI examinations (including up- and downgrade influences by DCE). Overall, the study’s cohort exhibited a wide range of image quality, where the majority achieved an overall PI-QUAL score of 2 or higher. Section S2 Figures 1–3 provide examples of T2-weighted (T2W), diffusion-weighed imaging (DWI), and dynamic-contrast enhanced (DCE) sequences as part of inadequate quality, acceptable quality and optimal quality examinations included in this study.

| **Section S2 Table 1 \|** PI-QUALv2 score for bpMRI and mpMRI | | |
| --- | --- | --- |
| **PI-QUAL score** | **bpMRI** | **mpMRI** |
| 1 (inadequate) | 174 (43%) | 139 (35%) |
| 2 (acceptable) | 160 (40%) | 240 (60%) |
| 3 (optimal) | 66 (17%) | 21 (5%) |
| bpMRI: biparametric MRI; mpMRI: multiparametric MRI (includes dynamic contrast-enhanced MRI) | | |


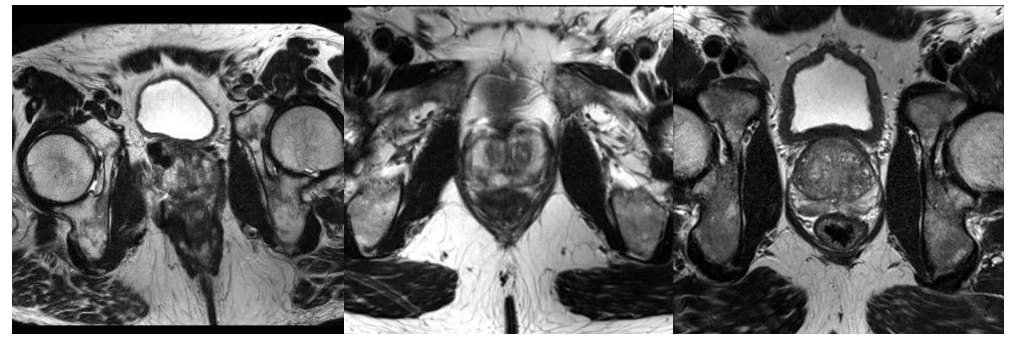


Section S2 Figure 1 – Three different axial T2-weighted sequences as part of examinations included in this study. (Left) Examination with inadequate T2W image quality according to PI-QUAL v2 exhibiting low signal-to-noise ratio, inability to delineate prostate structures, and movement artefacts in the prostate region; (Middle) Examination with degraded image quality due to minor movement artefacts and inability to delineate prostatic structures; (Right) Optimal axial T2W image quality.


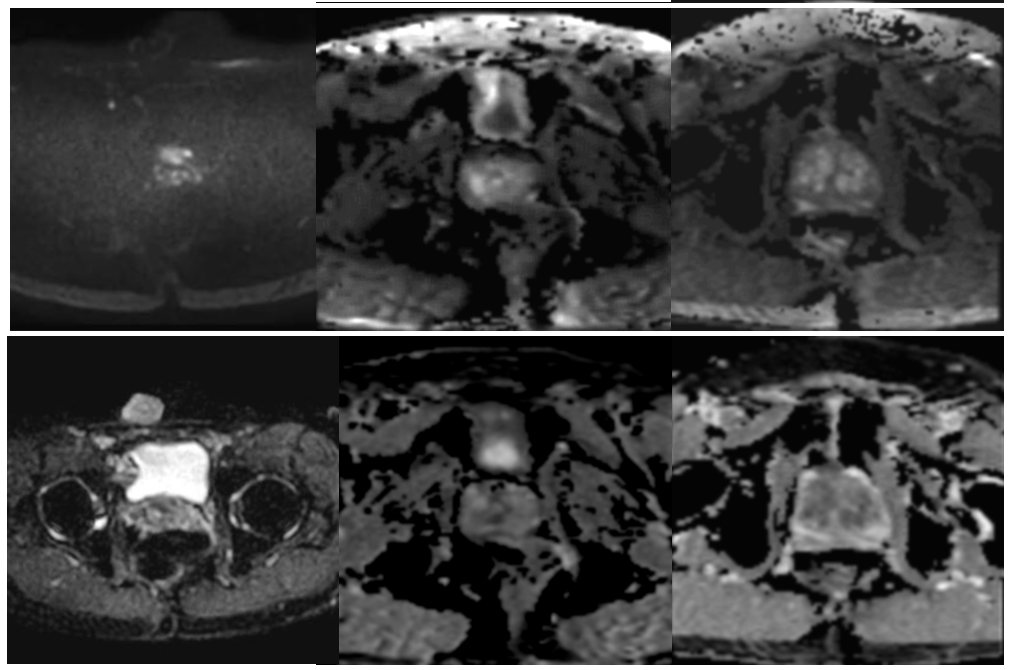


Section S2 Figure 2 – Three different diffusion-weighted imaging (DWI) with high b-value and apparent diffusion coefficient (ADC) map sequences as part of examinations included in this study. (Top and bottom left) Examination with inadequate DWI quality; this high b-value sequence shows low resolution, large field-of-view, and low signal-to-noise ratio, but no significant artifacts in the prostate region. On the ADC map, this exam shows a degraded image quality due to low signal-to-noise en minor susceptibility artefacts at the rectoprostatic interface; (Top and bottom middle) Examination with lower DWI quality due to the inability to discriminate the intraprostatic structures and lower ADC map quality due to minor susceptibility artefacts at rectoprostatic interface; (Top and bottom right) Examination with optimal DWI quality and ADC map quality.


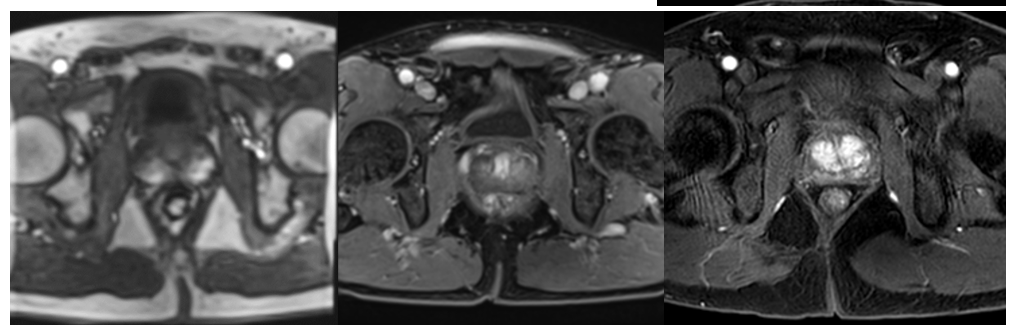


Section S2 Figure 3 – Three different dynamic contrast-enhanced (DCE) MRI sequences as part of examinations included in this study. (Left) Examination with inadequate image quality. The sequences is acquired without a fat-suppression technique, movement artefacts and the inability to identify anatomical structures. (Middle) Examination with lower image quality due to minimal movement artefacts leading to inability to clearly delineate intraprostatic structures; (Right) Examination with optimal DCE image quality.

[1] de Rooij M, Allen C, Twilt JJ, et al. PI-QUAL version 2: an update of a standardised scoring system for the assessment of image quality of prostate MRI. Eur Radiol 2024

**Section S3. Statistical Analysis Plan**

The statistical analysis plan was drafted in conjunction with the statistical analysis plan of the PI-CAI challenge. The plan was reviewed by the scientific advisory board of PI-CAI and a bio-statistician (N.A. Obuchowski) with expertise in diagnostic observer studies. The final document was dated and signed (as shown below). No changes were made in the final analysis of primary statistical tests, relative to the following prespecified plan.

**Statistical Analysis Plan**

The PI-CAI: Reader Study investigated clinically significant prostate cancer detection at biparametric MRI and at multiparametric MRI (including dynamic contrast enhanced MRI) within a multi-reader multi case (MRMC) observer study. The primary comparison within the PI-CAI: Reader Study involves clinically significant prostate cancer diagnostic performance between the two MRI protocols.

In this study, the primary outcomes are the patient-level sensitivity and specificity of clinically significant prostate cancer (defined as Gleason grade group 2 or greater) in men at a patient-level cutoff of PI-RADS ≥ 3, and the patient-level diagnostic performance evaluated with the area under the receiver operating characteristic curve (AUROC). Patient-level PI-RADS score is defined as the highest PI-RADS score within a case. Empirical receiver operating characteristic (ROC) curves are derived from the patient-level suspicion scores (ranging from 0 to 100). Multi-reader multi-case analysis of variance (ANOVA) using the Obuchowski-Rockette (OR)2 method is used to obtain diagnostic performances at PI-RADS ≥ 3 operating point, empirical area under the receiver operating characteristic curve (AUROC) values and corresponding 95% Wald confidence intervals and mean estimates. To construct the mean AUROC, the ANOVA-OR method estimates the AUROC for each reader, and then calculates the average.

For the primary outcomes, non-inferiority tests are performed using a non-inferiority margin of 0.05 considering a base alpha value (significance threshold) of 0.05. Non-inferiority of biparametric MRI is concluded if the lower boundary of the two-sided 95% or 97.5% confidence interval (pending correction for multiplicity) for the test statistic is greater than —0.05. To maintain the type I error while investigating multiple comparisons, all study objectives are prespecified in a hierarchical family tree and tested accordingly (as shown in Fig. 1).1 At each stage, the significance threshold and confidence intervals for the three primary endpoints are adjusted for multiplicity with the Holm-Bonferroni method.

For non-inferiority testing at PI-RADS ≥ 3 operating point, the test statistic is the reader sensitivity with biparametric MRI minus reader sensitivity with multiparametric MRI (Family 1A) and the reader specificity with biparametric MRI minus reader specificity with multiparametric MRI (Family 1B). The non-inferiority test with specificity as test statistic is only performed when non-inferior sensitivity is concluded. For non-inferiority testing with patient-level suspicion score, the test statistic is the reader AUROC with biparametric MRI minus the reader AUROC with multiparametric MRI. The non-inferiority margin was decided upon by the scientific advisory board of PI-CAI and in compliance with recent recommendations3 and the PRIME trial4, which investigate similar endpoints as this study.

Family 1A: Test the null hypothesis that radiologists with biparametric MRI are inferior to radiologists with multiparametric MRI at a cut-off of PI-RADS ≥ 3 in detecting clinically significant prostate cancer —using the difference in mean sensitivity across 400 cases. For this comparison, radiologists’ performance is derived from the PI-RADS scores reported during a MRMC observer study.

Family 1B: Test the null hypothesis that radiologists with biparametric MRI are inferior to radiologists with multiparametric MRI at case-level diagnosis of clinically significant prostate cancer —using the difference in mean AUROC across 400 cases. For this comparison, radiologists’ performance is derived from the case-level suspicion scores reported during a MRMC observer study.

**α/*k***

**= 0.025**

**α/(*k*–1)**

**= 0.050**

Family 2A: Test the null hypothesis that radiologists with biparametric MRI are inferior to radiologists with multiparametric MRI at a cut-off of PI-RADS ≥ 3 in ruling out clinically significant prostate cancer —using the difference in mean specificity across 400 cases. For this comparison, radiologists’ performance is derived from the PI-RADS scores reported during a MRMC observer study.

Figure 1: Flowchart illustrates the strategic plan to test study objectives while maintaining the type I error rate. Significance thresholds used for family 1A and 1B are adjusted using the Holm–Bonferroni method, considering a base alpha value of 0.05. Family 2A is only tested if the null hypothesis for family 1A is rejected. If the null hypotheses for both family 1A and 1B are rejected, family 2A is tested with an alpha value of 0.05. If the null hypothesis for family 1A is rejected, but that of family 1B is not rejected, then we move on to testing family 2A with an alpha value of 0.025.

**Section S3. Statistical Analysis Plan** *(continued)*

**Calculation of P Values for Non-Inferiority Tests**

The Obuchowski-Rockette (OR) model is designed for the non-equivalence setting. For non-inferiority, the exact P value can be derived from the ANOVA OR model components.^5^ The test statistic for non-inferiority, with $\hat{\theta}_{bpMRI\bullet}$ the estimated average reader AUROC for biparametric MRI, $\hat{\theta}_{bpMRI\bullet}$ the estimated average reader AUROC for multiparametric MRI and $\delta$ the prespecified non-inferiority margin is calculated with:

$$t= \frac{\hat{\theta}_{bpMRI\bullet}-\hat{\theta}_{mpMRI\bullet}+\delta}{\hat{s}}$$

where $\hat{s}$ is the estimated standard deviation of $\hat{\theta}_{bpMRI\bullet}-\hat{\theta}_{mpMRI\bullet}+\delta$, which is equal to the standard deviation of $\hat{\theta}_{bpMRI\bullet}-\hat{\theta}_{mpMRI\bullet}$ derived from the OR model. One can compute the P value using:

$$P= 2(1-F(t;{\hat{d}f}_{0}|H_{0})$$

where $F(t;{\hat{d}f}_{0}|H_{0})$ is the cumulative distribution function of the non-inferiority adjusted test statistic under the null hypothesis, which is a Student’s t distribution with ${\hat{d}f}_{0}$ degrees of freedom. The degrees of freedom are determined as:

$${\hat{d}f}_{0}=\frac{{\left\{ MS(T*R \right\}+H(J\left( {c\hat{o}v}_{2}-{c\hat{o}v}_{3} \right))\}}^{2}}{\left( MS\left( T*R \right) \right)^{2}/(J-1)}$$

where $MS\left( T*R \right)$ is the test-by-reader mean squares and ${c\hat{o}v}_{2}$ and ${c\hat{o}v}_{3}$ estimated covariances are obtained from the OR model, $J$ is the number of readers and $H\left( x \right)=x$ . ${\hat{d}f}_{0}$ is directly obtained from the OR model output.

**Software Packages**

Software for Obuchowski-Rockette MRMC analysis (MRMCaov v0.3.0) has been made publicly available by the University of Iowa under the following website: [**https://github.com/brian-j-smith/MRMCaov**](https://github.com/brian-j-smith/MRMCaov)**.**^6^

**References**

1. Obuchowski NA, Bullen J. Multireader diagnostic accuracy imaging studies: fundamentals of design and analysis. Radiology 2022; 303(1): 26-34.
2. Obuchowski NA, Rockette HE. Hypothesis testing of diagnostic accuracy for multiple readers and multiple tests: an ANOVA approach with dependent observations. Communications in Statistics-Simulation and Computation 1995; 24(2), 285-308.
3. Padhani AR, Schoots IG, Barentsz JO. Fast Magnetic Resonance Imaging as a Viable Method for Directing the Prostate Cancer Diagnostic Pathway, Eur. Urol. Oncol. 2021; 4(6): 863-865
4. Ng A, Khetrapal P, Kasivisvanathan V. Is it PRIME time for biparametric magnetic resonance imaging in prostate cancer diagnosis?. Eur Urol 2022; 82(1): 1-2.
5. Chen W, Petrick NA, Sahiner B. Hypothesis testing in noninferiority and equivalence MRMC ROC studies. Acad Radiol 2012; 19(9): 1158-1165.
6. Smith BJ, Hillis SL. Multi-reader multi-case analysis of variance software for diagnostic performance comparison of imaging modalities. Proceedings of SPIE 11316, Medical Imaging 2020: Image Perception, Observer Performance, and Technology Assessment, 113160K.


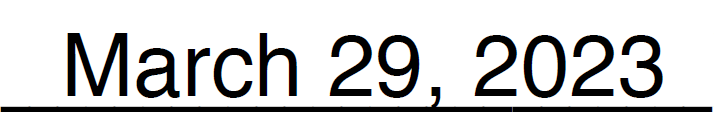


I, hereby, approve the aforementioned statistical analysis plan (dated ), as intended for analyzing the outcomes of the PI-CAI challenge (ClinicalTrials.gov identifier NCT05489341; BIAS preregistration https://zenodo.org/record/6667655).

**
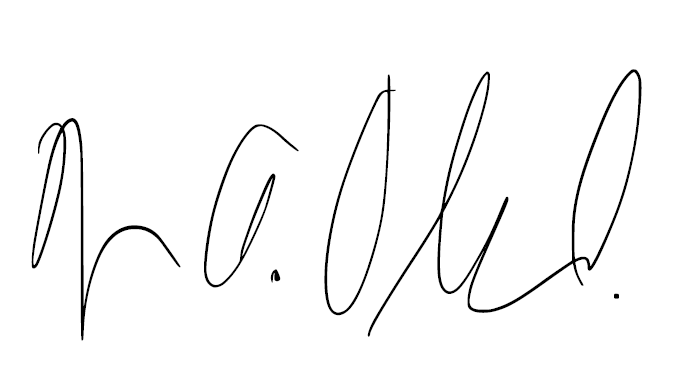
**

**_________________________________________________________________**

**Nancy A. Obuchowski, PhD**Department of Quantitative Health Sciences,
Cleveland Clinic Foundation,
9500 Euclid Ave, JJN3, Cleveland, OH 44195.

**Section S3. Supplementary Figures**


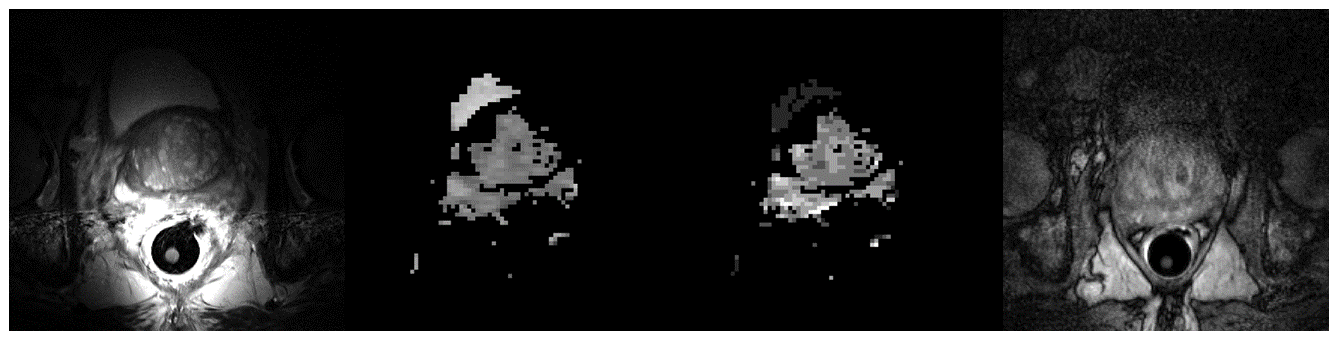


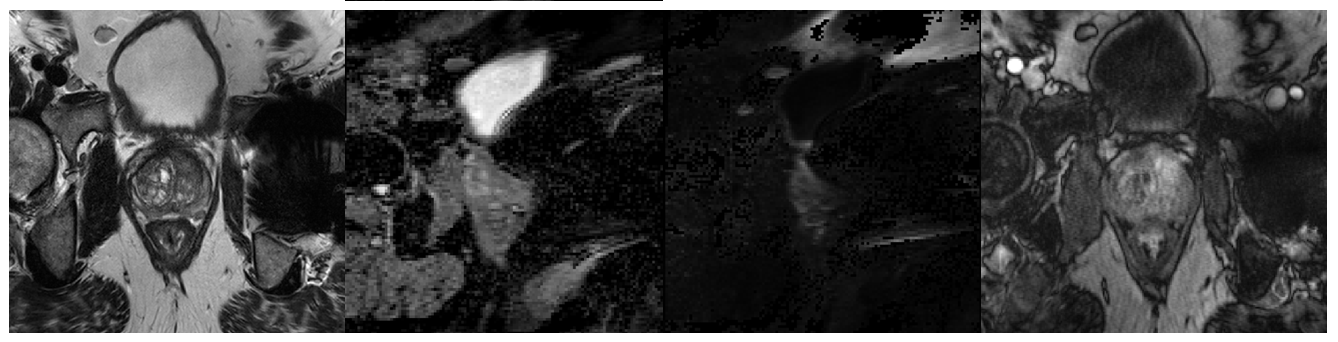


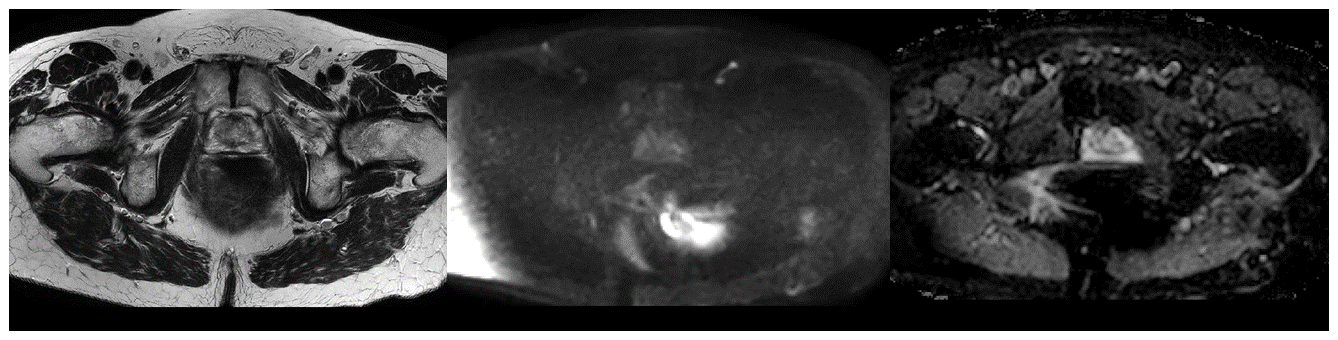


Supplementary Fig. 1 – Four different examples of excluded examinations due to severe artefacts. *Row 1*: From left to right, T2-Weighted (T2W) axial, diffusion weighted imaging (DWI) with high *b*-value, apparent diffusion coefficient (ADC) map, and dynamic contrast-enhanced (DCE) MRI. This examination is acquired using an endorectal coil and shows deformation of the posterior part of the prostate, low resolution on DWI and low signal-to-noise on DCE. *Row 2*: From left to right, T2W axial, DWI with high *b*-value, ADC map and DCE MRI. Specifically, DWI sequences are affected by the left hip prosthesis, which produces severe susceptibility artefacts across the left side of the prostate. *Row 3*: From left to right, T2W axial, DWI with high *b*-value, and ADC map. The DWI sequences are affected by severe susceptibility artefacts caused by rectal gas, most prominent in the dorsal part of the prostate at the rectoprostatic interface.

**Section S3. Supplementary Figures** *(continued)*


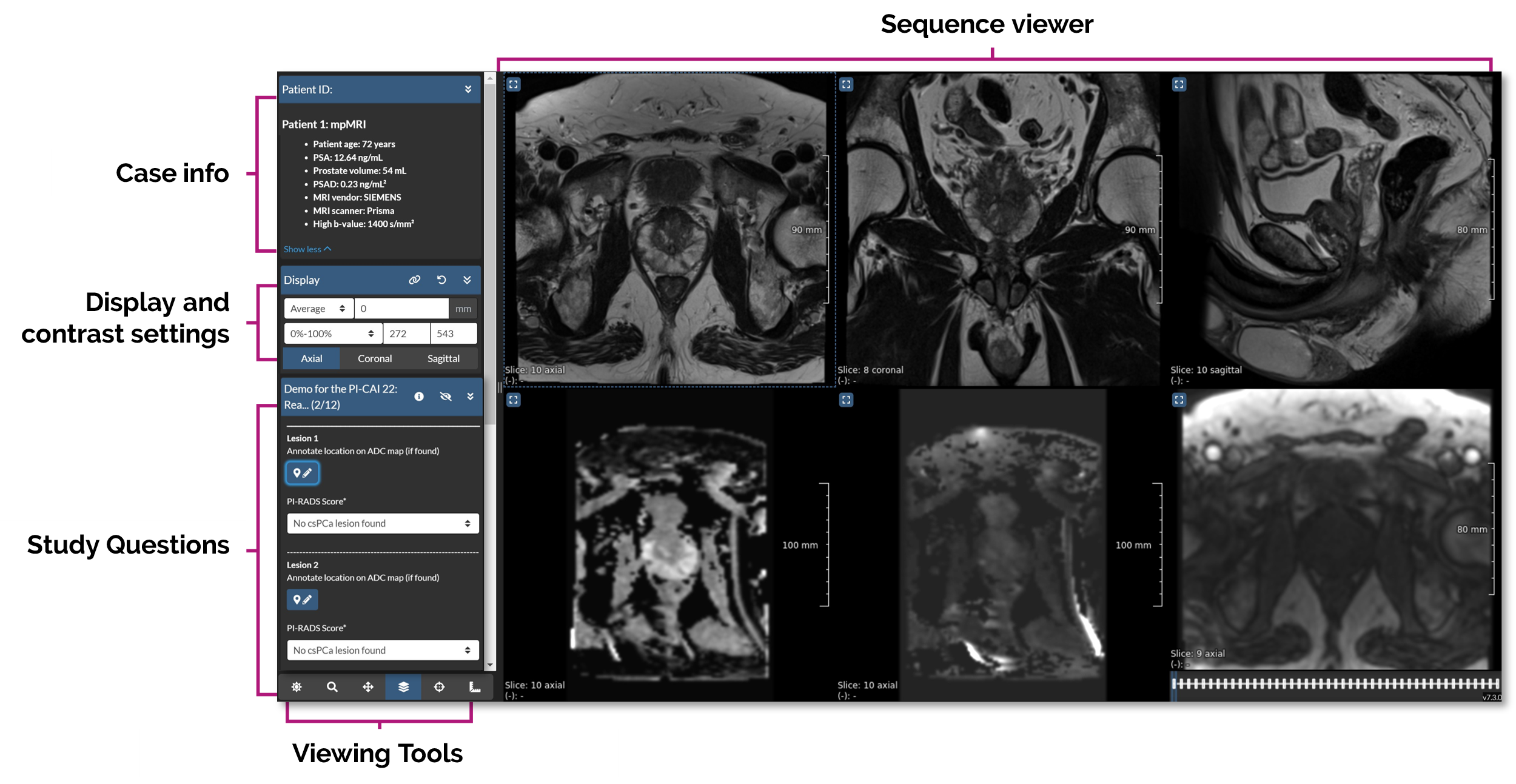


Supplementary Fig. 2 – Example of the workflow environment of the observer study for a multiparametric (mp)MRI examination. In the sequence viewer the full mpMRI protocol is available with from left to right in the top row T2-Weighted imaging in axial, coronal and sagittal orientations and in the second row diffusion weighted imaging (DWI) ADC-map, DWI high b-value and dynamic contrast-enhanced (DCE) MRI. For biparametric MRI examinations, DCE MRI was hidden. In the left upper corner, examination specific information, including clinical and MRI specific information was made available. The workstation had similar functionalities as a PACs system, where readers could change display and contrast settings, zoom, pan, link and scroll through sequences. In the left lower corner study questions were shown.

**Section S3. Supplementary Figures** *(continued)*

**
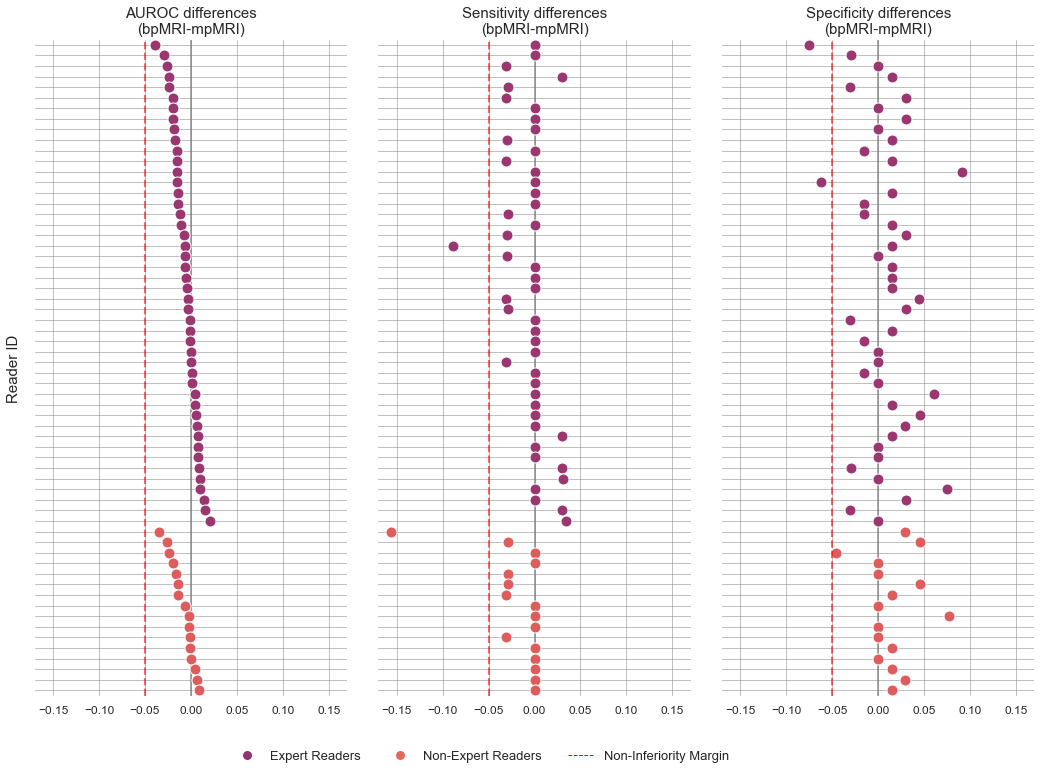
**

Supplementary Fig. 3 – Differences in diagnostic performances (biparametric (bp) MRI minus multiparametric (mp) MRI) per reader (n=62), considering the area under the operating characteristic curve (AUROC), sensitivity, and specificity at PI-RADS ≥ 3. Readers are categorized based on expert criteria (n=46) and non-expert criteria (n=16), following definitions set by ESUR/ESUI guidelines [1]. The non-inferiority margin was set at a 5% difference in diagnostic performance. For both reader groups, there was a lower variability in sensitivity differences as compared to specificity differences.

[1] de Rooij M, Israël B, Tummers M, et al. ESUR/ESUI consensus statements on multi-parametric MRI for the detection of clinically significant prostate cancer: quality requirements for image acquisition, interpretation and radiologists' training. Eur Radiol. 2020; 30(10): 5404-5416.

**Section S3. Supplementary Figures** *(continued)*


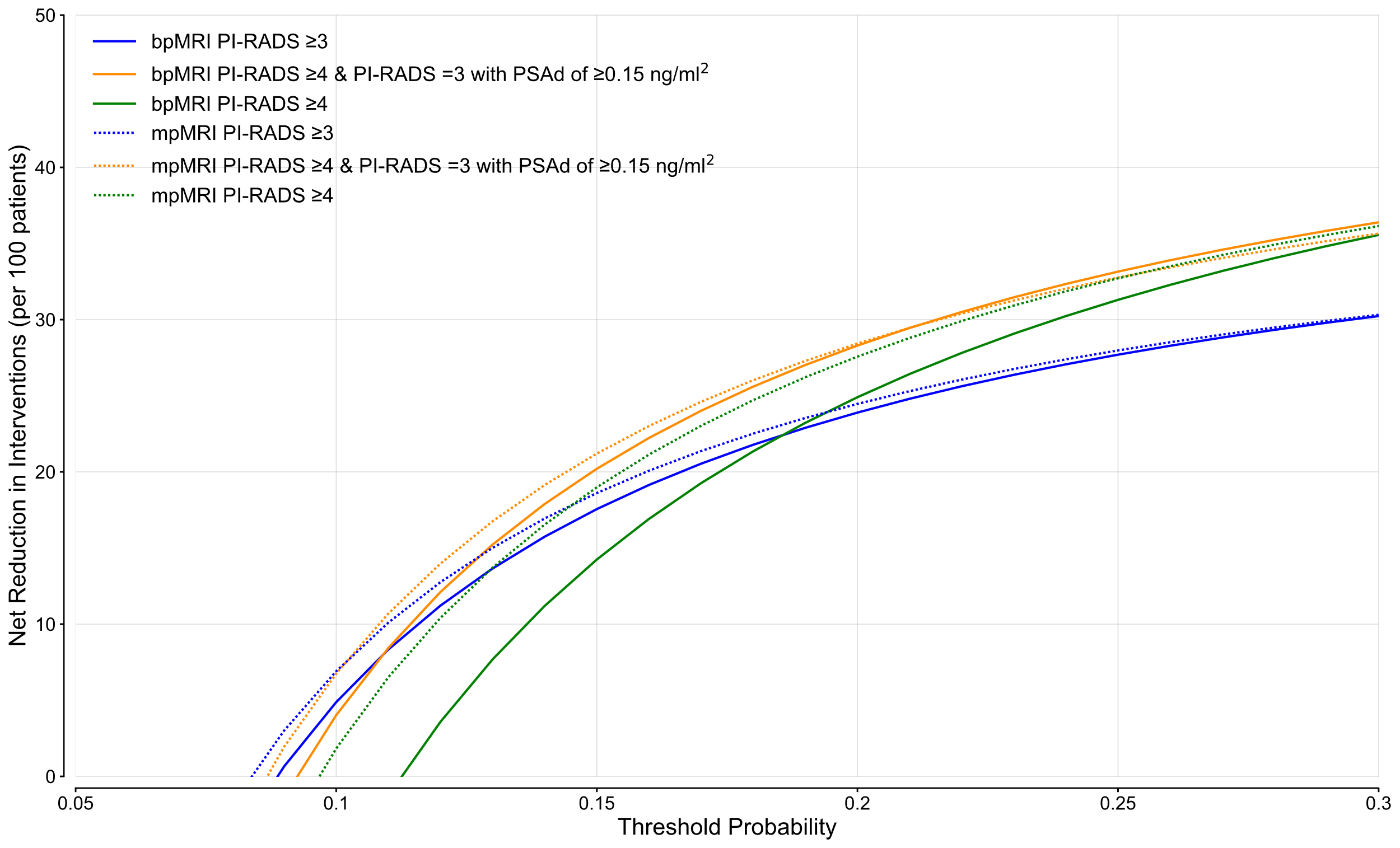


Supplementary Fig. 4 – Decision curve analysis for diagnosing clinically significant prostate cancer (csPCa) with biparametric (bp) and multiparametric (mp) MRI across three different risk thresholds, illustrating the net reduction in interventions per 100 patients. Threshold probabilities range from 5% to 30%, representing the willingness to conduct biopsies to diagnose one csPCa. Lower thresholds represent cancer averse scenarios while higher thresholds indicate biopsy averse scenarios. The net benefit is expressed in terms of true negatives relative to the biopsy-all pathway. To illustrate, at a threshold of 10% (willingness to perform ten biopsies to diagnose one csPCa), both the mpMRI at PI-RADS ≥3 and mpMRI at PI-RADS ≥4 & PI-RADS =3 with prostate-specific antigen density (PSAd) of ≥0.15 pathways are strategies that reduce the number of interventions by 7 per 100 patients without missing csPCa. Across thresholds, mpMRI at PI-RADS ≥3, mpMRI at PI-RADS ≥4 & PI-RADS =3 with prostate-specific antigen density (PSAd) of ≥0.15 ng/ml², as well as bpMRI at PI-RADS ≥4 & PI-RADS =3 with PSAd of ≥0.15 ng/ml², exhibited the highest net reduction in interventions. Differences in net reduction in interventions were small between pathways.

**Section S4. Supplementary Tables**

| **Supplementary Table 1 \|** MRI protocols | | | | |
| --- | --- | --- | --- | --- |
|  | **RUMC**  Nijmegen | **ZGT**   Twente | **PCNN**   Groningen | **OTUH**  Trondheim |
| **MRI manufacturer** | Siemens | Siemens | Siemens, Philips | Siemens |
| **MRI Model** | Prisma, Skyra | Skyra | Achieva, Aera, Avanto, Ingenia, Prisma, Skyra | Skyra |
| **Field Strength** (Tesla) | 3 | 3 | 1.5, 3 | 3 |
| **T2-weighted Imaging** |  |  |  |  |
| — Slice thickness (mm) | 3, 3.5, 4 | 1, 3, 3.5, 4 | 3 | 3 |
| — Voxel size (mm) | 0.3, 0.5 | 0.5-1.1 | 0.2, 0.3, 0.5, 0.6 | 0.5, 0.6 |
| **Diffusion Weighted Imaging** |  |  |  |  |
| — ADC b-values (s/mm^2^) | 0, 50;  50, 800 | 50, 800;  0, 1000 | 50, 1000; 50,400,800; 50,400,800,2000 | 0, 800 |
| — Calculated high b-value (s/mm^2^) | 1400 | 1400 | 1400, 2000 | 1400 |
| — Slice thickness (mm) | 3, 3.5, 4 | 3, 3.5, 4 | 3 | 3 |
| — Voxel size (mm) | 2 | 1.6, 1.7, 2 | 0.9, 1.4, 1.5, 1.8 | 2 |
| **Dynamic contrast-enhanced MRI** |  |  |  |  |
| — Gadolinium contrast | Dota, Gado | Dota | Dota, Gado | Dota |
| — Temporal resolution (s) | 3,4 | 3, 4 | 5,9,11 | 5, 6, 9 |
| — Slice Thickness (mm) | 3, 3.5 | 3 | 3 | 3 |
| — Voxel size (mm) | 0.8, 0.9 | 0.9 | 0.6, 1.4 | 1.4, 1.8 |
| RUMC: Radboud University Medical Center, Netherlands; ZGT: Ziekenhuisgroep Twente, Netherlands; PCNN: Prostaat Centrum Noord-Nederland, Netherlands; STOH: St. Olav’s Hospital, Trondheim University Hospital, Norway; ADC: Apparent Diffusion Coefficient; Dota: Dotarem; Gado: Gadolinium | | | | |

| **Supplementary Table 2 \|** Lesion-level characteristics of the observer study | | | | | |
| --- | --- | --- | --- | --- | --- |
| **Data** | **RUMC**  Nijmegen | **ZGT**   Twente | **PCNN**   Groningen | **STOH**  Trondheim | **Total** |
| **No. of Positive MRI Lesions**^§^ | **72** | **76** | **71** | **48** | **267** |
| **— PI-RADS 3 (%)** | **12 (17%)** | **14 (18%)** | **10 (14%)** | **11 (23%)** | **47 (18%)** |
| — in PZ (%) | 8 (67%) | 9 (64%) | 6 (60%) | 5 (45%) | 28 (60%) |
| — in TZ* (%) | 4 (33%) | 5 (36%) | 4 (40%) | 6 (55%) | 19 (40%) |
| **— PI-RADS 4 (%)** | **25 (35%)** | **28 (37%)** | **35 (49%)** | **14 (29%)** | **102 (38%)** |
| — in PZ (%) | 21 (84%) | 24 (86%) | 27 (77%) | 12 (86%) | 84 (82%) |
| — in TZ* (%) | 4 (16%) | 4 (14%) | 8 (23%) | 2 (14%) | 18 (18%) |
| **— PI-RADS 5 (%)** | **35 (49%)** | **34 (45%)** | **26 (37%)** | **23 (48%)** | **118 (44%)** |
| — in PZ (%) | 29 (83%) | 30 (88%) | 18 (69%) | 16 (70%) | 93 (79%) |
| — in TZ* (%) | 6 (17%) | 4 (12%) | 8 (31%) | 7 (30%) | 25 (21%) |
| **No. of GG Based Lesions** | **97** | **67** | **57** | **46** | **267** |
| — GG1 (%) | 48 (49%) | 32 (48%) | 21 (37%) | 7 (15%) | 108 (40%) |
| — GG2 (%) | 29 (30%) | 25 (37%) | 17 (30%) | 12 (26%) | 83 (31%) |
| — GG3 (%) | 10 (10%) | 4 (6%) | 13 (23%) | 16 (35%) | 43 (16%) |
| — GG4 (%) | 2 (2%) | 2 (3%) | 3 (5%) | 6 (13%) | 13 (5%) |
| — GG5 (%) | 8 (8%) | 4 (6%) | 3 (5%) | 5 (11%) | 20 (7%) |
| RUMC: Radboud University Medical Center, Netherlands; ZGT: Ziekenhuisgroep Twente, Netherlands; PCNN: Prostaat Centrum Noord-Nederland, Netherlands; STOH: St. Olav’s Hospital, Trondheim University Hospital, Norway; No.: Number; GG: Gleason Grade; PI-RADS: Prostate Imaging Reporting and Data System; PZ: Peripheral zone; TZ: Transition zone  ^§^ As determined by the original radiologist report from clinical routine  * Includes lesions (partly) located in central zone and anterior fibromuscular stroma | | | | | |

**Section S4. Supplementary Tables** *(continued)*

| **Supplementary Table 3 \|** Patient distribution and characteristics of the observer study per split-plot | | | | | | | | | | |
| --- | --- | --- | --- | --- | --- | --- | --- | --- | --- | --- |
|  | **Split-plot A** | | | | | **Split-plot B** | | | | |
|  | **RUMC**  Nijmegen | **ZGT**   Twente | **PCNN**   Groningen | **STOH**  Trondheim | **Total** | **RUMC**  Nijmegen | **ZGT**   Twente | **PCNN**   Groningen | **STOH**  Trondheim | **Total** |
| **No. of Sites** | **2** | **1** | **8** | **1** | **12** | **2** | **1** | **8** | **1** | **12** |
| **No. of MRI Scanners** | **2 S** | **1 S** | **2 S, 1 P** | **1 S** | **3 S, 1 P** | **2 S** | **1 S** | **2S, 1 P** | **1 S** | **3 S, 1 P** |
| **No. of Patients** | **33** | **26** | **21** | **20** | **100** | **33** | **27** | **20** | **20** | **100** |
| — Median Age (years) (IQR) | 65 (61-69) | 66 (56-71) | 64 (62-66) | 67 (62-72) | 65 (61-70) | 66 (56-69) | 65 (59-66) | 72 (63-74) | 64 (59-68) | 66 (59-70) |
| — Median PSA (ng/mL) (IQR) | 6.5 (5.4-8.8) | 6.5 (5.3-8.1) | 8.5 (6.1-12.3) | 7.835 (6.2-16.1) | 7.0 (5.6-11) | 6.2 (5.1-9.1) | 5.9 (4.4-7.2) | 9.4 (6.7-15.5) | 6.3 (5.0-9.6) | 6.6 (5.0-9.5) |
| — Median Prostate Volume (mL) (IQR) | 53 (42-82) | 50 (40-76) | 45 (32-61) | 48 (38-69) | 50 (40-70) | 63 (47-86) | 52 (41-81) | 51 (36-60) | 50 (31-79) | 55 (40-83) |
| — Median PSAd (ng/mL^2^) (IQR) | 0.11 (0.08-0.16) | 0.11 (0.08-0.20) | 0.2 (0.13-0.29) | 0.14 (0.1-0.35) | 0.13 (0.09-0.21) | 0.1 (0.09-0.15) | 0.11 (0.07-0.14) | 0.16 (0.14-0.29) | 0.11 (0.08-0.15) | 0.12 (0.09-0.16) |
| **No. of Benign or insignPCa cases (%)** | **24 (73%)** | **18 (67%)** | **12 (57%)** | **13 (65%)** | **67 (67%)** | **24 (73%)** | **19 (70%)** | **12 (60%)** | **13 (65%)** | **68 (68%)** |
| **No. of csPCa cases (%)** | **9 (27%)** | **8 (33%)** | **9 (43%)** | **7 (35%)** | **33 (33%)** | **9 (27%)** | **8 (30%)** | **8 (40%)** | **7 (35%)** | **32 (32%)** |
| — With Index lesion in PZ (%) | 9 (100%) | 7 (88%) | 7 (78%) | 5 (71%) | 28 (85%) | 7 (78%) | 8 (100%) | 6 (75%) | 4 (57%) | 25 (78%) |
| — With Index lesion in TZ* (%) | 0 (0%) | 1 (12%) | 2 (22%) | 2 (29%) | 5 (15%) | 2 (22%) | 0 (0%) | 2 (25%) | 3 (43%) | 7 (22%) |
| **No. of reference for patient** |  |  |  |  |  |  |  |  |  |  |
| — No hist. with follow-up (%) ^†^ | 15 (45%) | 0 (0%) | 0 (0%) | 5 (25%) | 20 (20%) | 13 (39%) | 0 (0%) | 0 (0%) | 6 (30%) | 19 (19%) |
| — Sys. TRUSBx (%) | 8 (24%) | 13 (50%) | 8 (38%) | 9 (45%) | 38 (38%) | 6 (18%) | 11 (41%) | 4 (20%) | 8 (40%) | 29 (29%) |
| — MRGBx (%) | 1 (3%) | 0 (0%) | 8 (38%) | 0 (0%) | 9 (9%) | 0 (0%) | 0 (0%) | 14 (70%) | 0 (0%) | 14 (14%) |
| — MRGBx + Sys. TRUS (%) | 8 (24%) | 8 (30%) | 0 (0%) | 1 (5%) | 17 (17%) | 10 (30%) | 9 (33%) | 0 (0%) | 2 (10%) | 21 (21%) |
| — Radical Prostatectomy (%) | 1 (3%) | 5 (20%) | 5 (24%) | 5 (25%) | 16 (16%) | 4 (12%) | 7 (26%) | 2 (10%) | 4 (20%) | 17 (17%) |
| **No. of ISUP scores** ^‡^ |  |  |  |  |  |  |  |  |  |  |
| — GG1 (%) | 3 (9%) | 6 (23%) | 4 (19%) | 0 (0%) | 13 (13%) | 5 (15%) | 9 (33%) | 4 (20%) | 1 (6%) | 19 (19%) |
| — GG2 (%) | 4 (12%) | 3 (12%) | 4 (19%) | 0 (0%) | 11 (11%) | 4 (12%) | 7 (26%) | 4 (20%) | 4 (20%) | 19 (19%) |
| — GG3 (%) | 1 (3%) | 3 (12%) | 3 (14%) | 3 (15%) | 10 (10%) | 4 (12%) | 0 (0%) | 3 (15%) | 2 (10%) | 9 (9%) |
| — GG4 (%) | 1 (3%) | 1 (4%) | 1 (5%) | 2 (10%) | 5 (5%) | 1 (3%) | 0 (0%) | 1 (5%) | 0 (0%) | 2 (2%) |
| — GG5 (%) | 3 (9%) | 1 (4%) | 1 (5%) | 2 (10%) | 7 (7%) | 0 (0%) | 1 (4%) | 0 (0%) | 1 (5%) | 2 (2%) |
| **No. PI-RADS scores** ^‡§^ |  |  |  |  |  |  |  |  |  |  |
| — PI-RADS 1-2 (%) | 23 (70%) | 13 (50%) | 5 (24%) | 13 (65%) | 54 (54%) | 19 (58%) | 12 (44%) | 2 (10%) | 10 (50%) | 43 (43%) |
| — PI-RADS 3 (%) | 1 (3%) | 1 (4%) | 4 (19%) | 0 (0%) | 6 (6%) | 1 (3%) | 1 (4%) | 2 (10%) | 2 (10%) | 6 (6%) |
| — PI-RADS 4 (%) | 3 (9%) | 6 (23%) | 10 (48%) | 0 (0%) | 19 (19%) | 6 (18%) | 8 (30%) | 8 (40%) | 2 (10%) | 24 (24%) |
| — PI-RADS 5 (%) | 6 (18%) | 6 (23%) | 2 (10%) | 7 (35%) | 21 (21%) | 7 (21%) | 6 (22%) | 8 (40%) | 6 (30%) | 27 (27%) |
| **No. of Positive MRI Lesions** |  |  |  |  |  |  |  |  |  |  |
| — PI-RADS 3 | 2 (15%) | 2 (12%) | 5 (28%) | 0 (0%) | 9 (16%) | 3 (15%) | 6 (25%) | 2 (10%) | 3 (25%) | 14 (18%) |
| — PI-RADS 4 | 4 (31%) | 9 (53%) | 11 (61%) | 2 (22%) | 26 (46%) | 8 (40%) | 11 (46%) | 11 (52%) | 3 (25%) | 33 (43%) |
| — PI-RADS 5 | 7 (54%) | 6 (35%) | 2 (11%) | 7 (78%) | 22 (39%) | 9 (45%) | 7 (29%) | 8 (38%) | 6 (50%) | 30 (39%) |
| **No. of ISUP-Based Lesions** |  |  |  |  |  |  |  |  |  |  |
| — GGG 1 | 6 (35%) | 9 (53%) | 5 (31%) | 0 (0%) | 20 (33%) | 14 (52%) | 14 (64%) | 6 (43%) | 2 (18%) | 36 (49%) |
| — GGG 2 | 6 (35%) | 3 (18%) | 5 (31%) | 0 (0%) | 14 (23%) | 8 (30%) | 7 (32%) | 4 (29%) | 6 (55%) | 25 (34%) |
| — GGG 3 | 1 (6%) | 3 (18%) | 3 (19%) | 5 (45%) | 12 (20%) | 4 (15%) | 0 (0%) | 3 (21%) | 2 (18%) | 9 (12%) |
| — GGG 4 | 1 (6%) | 1 (6%) | 2 (12%) | 3 (27%) | 7 (11%) | 1 (4%) | 0 (0%) | 1 (7%) | 0 (0%) | 2 (3%) |
| — GGG 5 | 3 (18%) | 1 (6%) | 1 (6%) | 3 (27%) | 8 (13%) | 0 (0%) | 1 (5%) | 0 (0%) | 1 (9%) | 2 (3%) |
| RUMC: Radboud University Medical Center, Netherlands; ZGT: Ziekenhuisgroep Twente, Netherlands; PCNN: Prostaat Centrum Noord-Nederland, Netherlands; STOH: St. Olav’s Hospital, Trondheim University Hospital, Norway; No.: Number; IQR: Interquartile range; PSA: Prostate-specific Antigen; PSAd: Prostate-specific Antigen Density; insignPCa: Clinically insignificant prostate cancer (GG=1); csPCa: Clinically significant prostate cancer (GG≥2); PZ: Peripheral Zone; TZ: Transition Zone; Hist.: Histopathology; Sys TRUSBx: Systematic Transurectal Ultrasound Guided Biopsy; MRGBx: MRI Guided Biopsy; ISUP: International Society of Urological Pathology; GG: Gleason Grade; PI-RADS: Prostate Imaging Reporting and Data System; S: Siemens Healthineers MRI scanner; P: Phillips Medical Systems MRI scanner;  * Includes lesions (partly) located in central zone and anterior fibromuscular stroma  ^†^ Follow-up period of at least 3 years  ^‡^ Defined as the highest score found on a per-patient level; ^§^: As determined by the original radiologist report from clinical routine | | | | | | | | | | |

**Section S4. Supplementary Tables** *(continued)*

| **Supplementary Table 3** *(continued)* **\|**  Patient distribution and characteristics of the reader study blocks | | | | | | | | | | |
| --- | --- | --- | --- | --- | --- | --- | --- | --- | --- | --- |
| **Data Source** | **Split-plot C** | | | | | **Split-plot D** | | | | |
|  | **RUMC**  Nijmegen | **ZGT**   Twente | **PCNN**   Groningen | **STOH**  Trondheim | **Total** | **RUMC**  Nijmegen | **ZGT**   Twente | **PCNN**   Groningen | **STOH**  Trondheim | **Total** |
| **No. of Sites** | **2** | **1** | **8** | **1** | **12** | **2** | **1** | **8** | **1** | **12** |
| **No. of MRI Scanners** | **2 S** | **1 S** | **2 S, 1 P** | **1 S** | **3 S, 1 P** | **2 S** | **1 S** | **2 S, 1 P** | **1S** | **3 S, 1 P** |
| **No. of Patients** | **34** | **26** | **20** | **20** | **100** | **35** | **27** | **18** | **20** | **100** |
| — Median Age (years) (IQR) | 64 (59-68) | 64 (61-68) | 70 (65-74) | 68 (65-70) | 66 (62-69) | 65 (58-68) | 64 (56-68) | 65 (64-69) | 67 (56-72) | 65 (58-68) |
| — Median PSA (ng/mL) (IQR) | 7.8 (5.5-9.8) | 6.4 (5.5-8.8) | 10.5 (7.9-20.2) | 7.4 (6.2-9.1) | 7.8 (5.7-10.5) | 7.2 (4.7-9.6) | 7.0 (5.6-9.5) | 9.0 (6.9-13.7) | 6.8 (5.4-9.3) | 7.2 (5.5-9.9) |
| — Median Prostate Volume (mL) (IQR) | 64 (43-82) | 50 (37-72) | 49 (35-80) | 65 (47-71) | 60 (40-79) | 60 (49-84) | 59 (39-73) | 50 (35-63) | 44 (35-57) | 54 (38-75) |
| — Median PSAd (ng/mL^2^) (IQR) | 0.12 (0.09-0.18) | 0.13 (0.08-0.19) | 0.22 (0.16-0.34) | 0.11 (0.08-0.25) | 0.14 (0.09-0.22) | 0.11 (0.08-0.15) | 0.12 (0.08-0.22) | 0.19 (0.14-0.27) | 0.13 (0.11-0.18) | 0.13 (0.09-0.21) |
| **No. of Benign or insignPCa cases (%)** | **24 (71)** | **17 (66)** | **12 (60)** | **13 (65)** | **66 (66)** | **24 (69)** | **19 (70)** | **10 (55)** | **13 (65)** | **66 (66)** |
| **No. of csPCa cases (%)** | **10 (29)** | **9 (34)** | **8 (40)** | **7 (35)** | **34 (34)** | **11 (31)** | **8 (30)** | **8 (45)** | **7 (35)** | **34 (34)** |
| — With Index lesion in PZ (%) | 9 (90) | 8 (89) | 7 (88) | 6 (86) | 30 (88) | 10 (90) | 7 (88) | 7 (88) | 6 (86) | 30 (88) |
| — With Index lesion in TZ* (%) | 1 (10) | 1 (11) | 1 (12) | 1 (14) | 4 (12) | 1 (10) | 1 (12) | 1 (12) | 1 (14) | 4 (12) |
| **No. of reference for patient** |  |  |  |  |  |  |  |  |  |  |
| — No hist. with follow-up (%) ^†^ | 12 (35) | 0 (0) | 0 (0) | 4 (2) | 16 (16) | 15 (43) | 0 (0) | 0 (0) | 2 (10) | 17 (17) |
| — Sys. TRUSBx (%) | 5 (15) | 14 (54) | 7 (35) | 11 (55) | 37 (37) | 5 (14) | 13 (48) | 5 (28) | 11 (55) | 34 (34) |
| — MRGBx (%) | 3 (9) | 0 (0) | 12 (60) | 0 (0) | 15 (15) | 5 (14) | 0 (0) | 9 (50) | 0 (0) | 14 (14) |
| — MRGBx + Sys. TRUS (%) | 11 (32) | 7 (27) | 0 (0) | 2 (1) | 20 (20) | 8 (23) | 8 (30) | 0 (0) | 4 (20) | 20 (20) |
| — Radical Prostatectomy (%) | 3 (9) | 5 (19) | 1 (5) | 3 (2) | 12 (12) | 2 (6) | 6 (22) | 4 (22) | 3 (15) | 15 (15) |
| **No. of ISUP scores** ^‡^ |  |  |  |  |  |  |  |  |  |  |
| — GG1 (%) | 6 (18) | 3 (12) | 4 (20) | 0 (0) | 13 (13) | 2 (6) | 5 (19) | 4 (22) | 2 (10) | 13 (13) |
| — GG2 (%) | 6 (18) | 7 (27) | 4 (20) | 2 (10) | 19 (19) | 6 (17) | 6 (22) | 3 (17) | 3 (15) | 18 (18) |
| — GG3 (%) | 2 (6) | 1 (4) | 3 (15) | 4 (20) | 10 (10) | 3 (9) | 0 (0) | 4 (22) | 2 (10) | 9 (9) |
| — GG4 (%) | 0 (0) | 0 (0) | 0 (0) | 1 (5) | 1 (1) | 0 (0) | 1 (4) | 0 (0) | 1 (5) | 2 (2) |
| — GG5 (%) | 2 (6) | 1 (4) | 1 (5) | 0 (0) | 4 (4) | 2 (6) | 1 (4) | 1 (6) | 1 (5) | 5 (5) |
| **No. PI-RADS scores** ^‡§^ |  |  |  |  |  |  |  |  |  |  |
| — PI-RADS 1-2 (%) | 19 (56) | 14 (54) | 3 (15) | 9 (45) | 45 (45) | 21 (60) | 13 (48) | 6 (33) | 12 (60) | 52 (52) |
| — PI-RADS 3 (%) | 2 (6) | 2 (8) | 1 (5) | 4 (20) | 9 (9) | 1 (3) | 2 (7) | 2 (11) | 2 (10) | 7 (7) |
| — PI-RADS 4 (%) | 5 (15) | 1 (4) | 5 (25) | 2 (10) | 13 (13) | 3 (9) | 3 (11) | 5 (28) | 3 (15) | 14 (14) |
| — PI-RADS 5 (%) | 8 (24) | 9 (35) | 11 (55) | 5 (25) | 33 (33) | 10 (29) | 9 (33) | 5 (28) | 3 (15) | 27 (27) |
| **No. of Positive MRI Lesions** |  |  |  |  |  |  |  |  |  |  |
| — PI-RADS 3 (%) | 3 (18) | 4 (21) | 1 (5) | 6 (35) | 14 (19) | 4 (18) | 2 (13) | 2 (17) | 2 (20) | 10 (17) |
| — PI-RADS 4 (%) | 6 (35) | 4 (21) | 8 (40) | 4(24) | 22 (30) | 7 (32) | 4 (25) | 5 (42) | 5 (50) | 21 (35) |
| — PI-RADS 5 (%) | 8 (47) | 11 (58) | 11 (55) | 7 (41) | 37 (51) | 11 (50) | 10 (62) | 5 (42) | 3 (30) | 29 (48) |
| **No. of ISUP-Based Lesions** |  |  |  |  |  |  |  |  |  |  |
| — GG1 (%) | 15 (60) | 3 (21) | 6 (40) | 1 (8) | 25 (38) | 13 (46) | 6 (43) | 4 (33) | 4 (33) | 27 (41) |
| — GG2 (%) | 6 (24) | 9 (64) | 5 (33) | 3 (25) | 23 (35) | 9 (32) | 6 (43) | 3 (25) | 3 (25) | 21 (32) |
| — GG3 (%) | 2 (8) | 1 (7) | 3 (20) | 6 (50) | 12 (18) | 3 (11) | 0 (0) | 4 (33) | 3 (25) | 10 (15) |
| — GG4 (%) | 0 (0) | 0 (0) | 0 (0) | 2 (17) | 2 (3) | 0 (0) | 1 (7) | 0 (0) | 1 (8) | 2 (3) |
| — GG5 (%) | 2 (8) | 1 (7) | 1 (7) | 0 (0) | 4 (6) | 3 (11) | 1 (7) | 1 (8) | 1 (8) | 6 (9) |
| RUMC: Radboud University Medical Center, Netherlands; ZGT: Ziekenhuisgroep Twente, Netherlands; PCNN: Prostaat Centrum Noord-Nederland, Netherlands; STOH: St. Olav’s Hospital, Trondheim University Hospital, Norway; No.: Number; IQR: Interquartile range; PSA: Prostate-specific Antigen; PSAd: Prostate-specific Antigen Density; insignPCa: Clinically insignificant prostate cancer (GG=1); csPCa: Clinically significant prostate cancer (GG≥2); PZ: Peripheral Zone; TZ: Transition Zone; Hist.: Histopathology; Sys TRUSBx: Systematic Transurectal Ultrasound Guided Biopsy; MRGBx: MRI Guided Biopsy; ISUP: International Society of Urological Pathology; GG: Gleason Grade; PI-RADS: Prostate Imaging Reporting and Data System; S: Siemens Healthineers MRI scanner; P: Phillips Medical Systems MRI scanner;  * Includes lesions (partly) located in central zone and anterior fibromuscular stroma  ^†^ Follow-up period of at least 3 years  ^‡^ Defined as the highest score found on a per-patient level; ^§^: As determined by the original radiologist report from clinical routine | | | | | | | | | | |

| **Supplementary Table 4 \|** Zonal distribution of patient-level PI-RADS scores at bpMRI and mpMRI | | | | |
| --- | --- | --- | --- | --- |
|  | **bpMRI P<3 – mpMRI P<3** | **bpMRI P<3 – mpMRI P3** | **bpMRI P<3 – mpMRI P4** | **bpMRI P<3 – mpMRI P5** |
| **Total (%)** | **2508 (41%)** | **57 (1%)** | **77 (1%)** | **9 (<1%)** |
| **— in PZ (%)** | - | 32 (56%) | 61 (22%) | 2 (22%) |
| **— in TZ* (%)** | - | 25 (44%) | 16 (78%) | 7 (78%) |
|  | **bpMRI P3 – mpMRI P<3** | **bpMRI P3 – mpMRI P3** | **bpMRI P3 – mpMRI P4** | **bpMRI P3 – mpMRI P5** |
| **Total (%)** | **57 (1%)** | **590 (10%)** | **206 (3%)** | **10 (<1%)** |
| **— in PZ (%)** | 42 (74%) | 300 (51%) | 168 (82%) | 3 (30%) |
| **— in TZ* (%)** | 15 (26%) | 290 (49%) | 38 (18%) | 7 (70%) |
|  | **bpMRI P4 – mpMRI P<3** | **bpMRI P4 – mpMRI P3** | **bpMRI P4 – mpMRI P4** | **bpMRI P4 – mpMRI P5** |
| **Total (%)** | **23 (<1%)** | **39 (1%)** | **1092 (18%)** | **37 (1%)** |
| **— in PZ (%)** | 15 (65%) | 23 (59%) | 813 (74%) | 25 (68%) |
| **— in TZ* (%)** | 8 (35%) | 16 (41%) | 279 (26%) | 12 (32%) |
|  | **bpMRI P5 – mpMRI P<3** | **bpMRI P5 – mpMRI P3** | **bpMRI P5 – mpMRI P4** | **bpMRI P5 – mpMRI P5** |
| **Total (%)** | **8 (<1%)** | **11 (<1%)** | **10 (<1%)** | **1440 (23%)** |
| **— in PZ (%)** | 4 (50%) | 7 (64%) | 9 (90%) | 747 (52%) |
| **— in TZ* (%)** | 4 (50%) | 4 (36%) | 1 (10%) | 693 (48%) |
| The table presents the zonal distribution of patient-level PI-RADS scores assigned during bpMRI and mpMRI assessments, categorizing them by annotations made within the peripheral zone (PZ) and transition zone (TZ). It underscores intra-reader consistency and highlights up- or downgrades between the different reading configurations by zone. The table is organized according to the four possible PI-RADS scores determined during the bpMRI assessment, with columns representing the corresponding PI-RADS score assigned at mpMRI.  bpMRI: biparametric MRI; mpMRI: multiparametric MRI; P: PI-RADS score; PZ: Peripheral zone; TZ: Transition zone  * Includes lesions (partly) located in central zone and anterior fibromuscular stroma | | | | |
